# Evolution and expression analysis of the grape (*Vitis vinifera* L.) *WRKY* gene family

**DOI:** 10.1093/jxb/eru007

**Published:** 2014-02-07

**Authors:** Chunlei Guo, Rongrong Guo, Xiaozhao Xu, Min Gao, Xiaoqin Li, Junyang Song, Yi Zheng, Xiping Wang

**Affiliations:** ^1^Key Laboratory of Horticultural Plant Biology and Germplasm Innovation in Northwest China, Ministry of Agriculture, Northwest A&F University, Yangling, Shaanxi 712100, China; ^2^State Key Laboratory of Crop Stress Biology in Arid Areas, College of Horticulture, Northwest A&F University, Yangling, Shaanxi 712100, China; ^3^Boyce Thompson Institute for Plant Research, Cornell University, Ithaca, NY 14853, USA

**Keywords:** Evolution, expression profile analysis, grape (*Vitis vinifera* L.), phylogenetic analysis, synteny analysis, *WRKY* genes.

## Abstract

WRKY proteins comprise a large family of transcription factors that play important roles in plant defence regulatory networks, including responses to various biotic and abiotic stresses. To date, no large-scale study of *WRKY* genes has been undertaken in grape (*Vitis vinifera* L.). In this study, a total of 59 putative grape *WRKY* genes (*VvWRKY*) were identified and renamed on the basis of their respective chromosome distribution. A multiple sequence alignment analysis using all predicted grape *WRKY* genes coding sequences, together with those from *Arabidopsis thaliana* and tomato (*Solanum lycopersicum*), indicated that the 59 *VvWRKY* genes can be classified into three main groups (I–III). An evaluation of the duplication events suggested that several *WRKY* genes arose before the divergence of the grape and *Arabidopsis* lineages. Moreover, expression profiles derived from semiquantitative PCR and real-time quantitative PCR analyses showed distinct expression patterns in various tissues and in response to different treatments. Four *VvWRKY* genes showed a significantly higher expression in roots or leaves, 55 responded to varying degrees to at least one abiotic stress treatment, and the expression of 38 were altered following powdery mildew (*Erysiphe necator*) infection. Most *VvWRKY* genes were downregulated in response to abscisic acid or salicylic acid treatments, while the expression of a subset was upregulated by methyl jasmonate or ethylene treatments.

## Summary

Fifty-nine *VvWRKY* genes were identified. Phylogenetic tree and synteny analysis revealed the specific evolutionary relationship of these genes. Meanwhile, differential expression patterns indicated their possible roles in specific tissues and under different stresses.

## Introduction

Transcription factors are proteins that bind to specific DNA sequences in the promoter regions of genes, thereby regulating their transcription. Consequently, transcription factors play pivotal roles in numerous plant signalling and regulatory networks (Hwang *et al.*, 2010). WRKY proteins, which are characterized by a highly conserved domain of about 60 amino acid residues, comprise a class of transcription factors that are known to function as transcriptional activators or repressors in a number of developmental and physiological processes (Eulgem *et al.*, 2000; Rushton *et al.*, 2010, 2012). The first *WRKY* gene to be cloned and characterized was from sweet potato (Ishiguro and Nakamura, 1994) and this was followed by studies of *WRKY* genes from *Arabidopsis* (Eulgem *et al.*, 2000), rice (Wu *et al.*, 2005), and barley (Mangelsen *et al.*, 2008). Moreover, large-scale genome-wide studies of *WRKY* genes have been described for *Arabidopsis* (Dong *et al.*, 2003), rice (Ryu *et al.*, 2006), poplar (He *et al.*, 2012), tomato (Huang *et al.*, 2012), cucumber (Ling *et al.*, 2011), and coffee (Ramiro *et al.*, 2010).

The two most prominent and defining structural characteristics of WRKY proteins are the WRKY domains and the zinc-finger motifs (C–X_4–5_–X_22–23_–H–X_1_–H or C–X_7_–C–X_23_–H–X_1_–C), which provide the basis of their classification into groups I–III (Eulgem *et al.*, 2000). Proteins from group I contain two WRKY domains and a C_2_H_2_ zinc-finger motif, while those from groups II and III only contain one WRKY domain and a C_2_H_2_ or C_2_HC zinc-finger motif, respectively (Eulgem *et al.*, 2000; Zhang and Wang, 2005). WRKY proteins bind to the W-box ((C/T)TGAC(T/C)) of their target genes (Ciolkowski *et al.*, 2008) as well as a *cis*-element (SURE), which plays a role in promoting transcription (Sun *et al.*, 2003).

WRKY transcription factors are involved in regulatory processes mediated by various biotic and abiotic stresses (Eulgem and Somssich, 2007; Pandey and Somssich, 2009). For example, defence responses in *Arabidopsis* to the bacterial pathogen *Pseudomonas syringae* have been associated with the action of *AtWRKY38* and *AtWRKY62* (Journot-Catalino *et al.*, 2006; Kim *et al.*, 2008) and the involvement of *WRKY* genes in the resistance of grapevine to pathogens was suggested by studies using transgenic tobacco lines expressing *VvWRKY2*, reflecting the important function of *WRKY* genes in biotic stress tolerance (Mzid *et al.*, 2007). *WRKY* genes are also related to abiotic stress induced gene expression: *AtWRKY25* and *AtWRKY33* show altered expression following either heat (Li *et al.*, 2009; Li *et al.*, 2011) or salt treatments (Jiang and Deyholos, 2009) and the expression of *TcWRKY53* from alpine pennygrass (*Thlaspi caerulescens*) is affected by cold, salt, and polyethylene glycol treatments (Rushton *et al.*, 2010). Studies involving other plants, including rice (Shimono *et al.*, 2011), wheat (Wu *et al.*, 2008) and poplar (He *et al.*, 2012), have similarly provided insights into the diversity of *WRKY* gene function.

Grape (*Vitis vinifera* L.) is used both as a fresh food commodity and for processed food products such as juice, raisins, and wine. It is cultivated worldwide and has great economic value (Kreamer, 1995; Jaillon *et al.*, 2007) and so there is considerable interest in identifying genes to improve grape horticultural characteristics, such as those that promote stress resistance. In this regard, the various studies of *WRKY* genes from different plant species mentioned above suggest that members of the grape *WRKY* gene family (*VvWRKY*) have considerable potential for contributing to aspects of stress resistance. This work reports the identification of 59 putative *VvWRKY* genes, together with an analysis of their exon–intron organization and associated gene duplication events in the context of gene evolution, since it has been shown that gene duplication has played a critical role in *Arabidopsis* and rice *WRKY* gene family expansion (Cannon *et al.*, 2004). In addition, this work determined the expression profiles of *VvWRKY* genes in six different tissues and measured their transcript abundance in response to different phytohormone treatments and under various abiotic and biotic stresses. This study provides a foundation for future studies of *VvWRKY* gene family evolution and function.

## Materials and methods

### Identification and annotation of grape *WRKY* genes

An Hidden Markov Model profile of the WRKY DNA-binding domain (PF03106) was downloaded from the Pfam protein family database (http://pfam.sanger.ac.uk/) (Finn *et al.*, 2010) and used to identify putative WRKY genes/proteins from the grape genome sequence (http://www.genoscope.cns.f) (Jaillon *et al.*, 2007) using the BLASTP program and default parameters (Ling *et al.*, 2011). All non-redundant gene sequences encoding complete WRKY domains were selected as putative *WRKY* genes. Expressed sequence tags (ESTs) of each gene sequence from the public *V. vinifera* EST database were used for further validation. The identified grape *WRKY* genes were annotated based on their respective chromosome distribution (Ling *et al.*, 2011).

### Multiple sequence alignment, phylogenetic analysis, and classification of grape *WRKY* genes

A total of 59 predicted VvWRKY proteins, with amino acids spanning the WRKY core domain, were included in multiple sequence alignments using CLUSTALX version 2.0.12 (Larkin *et al.*, 2007) and Boxshade (http://www.ch.embnet.org/software/BOX_form.html). A further multiple sequence alignment including *VvWRKY* genes and those from *Arabidopsis* (*AtWRKY*) and tomato (*Solanum lycopersicum*, *SlWRKY*) was performed using CLUSTALW. A phylogenetic tree based on the alignment was constructed using MEGA 5.0 with the neighbour-joining method and with the bootstrap test replicated 1000 times (Tamura *et al.*, 2011). Based on the multiple sequence alignment and the previously reported classification of *AtWRKY* genes, the *VvWRKY* genes were assigned to different groups and subgroups.

### Exon–intron structure, tandem duplication, and synteny analysis of grape *WRKY* genes

The exon–intron structures of the grape *WRKY* genes were determined based on alignments of their coding sequences and their respective full-length sequences (Grape Genome Browser; http://www.genoscope.cns.fr/externe/GenomeBrowser/Vitis/), while diagrams were obtained from the online program Gene Structure Display Server (GSDS: http://gsds.cbi.pku.edu.ch). *WRKY* genes with tandem duplication events were defined as adjacent homologous genes on a single chromosome, while gene duplication events between different chromosomes were characterized as segmental duplications (Liu *et al.*, 2011). The specific physical location of each *VvWRKY* gene on its individual chromosome therefore determined whether it was regarded as a genes resulting from a tandem duplication event. The syntenic blocks used for constructing a synteny analysis map of the grape *WRKY* genes, as well as between grape and *Arabidopsis WRKY* genes, were obtained from the Plant Genome Duplication Database (Tang *et al.*, 2008) and the diagrams were generated by the program Circos version 0.63 (http://circos.ca/).

### Plant material and treatments

Grape organs/tissues (roots, tendrils, leaves, inflorescences, fruit, and stems) were obtained from 2-year-old ‘Kyoho’ (*Vitis labrusca* × *V. vinifera*) grape seedlings, which were grown in 12 dm^3^ pots in the greenhouse of Northwest A&F University, Yangling, Shaanxi, China (34° 20′ N 108° 24′ E). The third to fifth fully expanded young grapevine leaves beneath the apex were taken for hormone treatments, at which time the shoots of the vines were 25–35cm long.

Hormone treatments were carried out by spraying leaves with 300 μM abscisic acid (ABA), 100 μM salicylic acid (SA), 50 μM methyl jasmonate (MeJA), and 0.5g/l ethylene (Eth), and then leaves were sampled at 0.5, 1, 6, 24, and 48h post treatment (Li *et al.*, 2010). Leaves sprayed with sterile water and similarly harvested were used as a negative control. A salinity stress treatment was carried out by irrigating plants with 2 l of 250mM NaCl in the pots (Upreti and Murti, 2010) followed by sampling leaves at 1, 6, 24, and 48h post treatment. Seedlings irrigated with 2 l tap water were used as a negative control. A drought stress treatment was performed by withholding water (Yang *et al.*, 2012) followed by sampling at 24, 48, 96, 144, and 168h post treatment. Plants were rewatered after 168h of drought stress and sampled again 48h later. Grape seedlings grown without drought stress were used as a control.

Samples of the powdery mildew fungus (*Erysiphe necator*) were used to inoculate young leaves of *V. quinquangularis* ‘Shang-24’ (Northwest A&F University, Yangling, Shanxi, China; Wang *et al.*, 1995). Leaves were harvested at 6, 12, 24, 48, 72, 96, and 120h post inoculation and uninoculated leaves served as a negative control.

At each time point of each treatment, six leaves from six separate plants were combined to form one sample, and all of the treatment experiments were performed in triplicate. All these plant samples were immediately frozen in liquid nitrogen and stored at –80°C until RNA extraction.

### Semiquantitative reverse-transcription PCR analysis

Total RNA samples were extracted from leaves using the EZNA Plant RNA Kit (R6827-01, Omega Bio-tek, USA). First-strand cDNA was synthesized by reverse transcription of 500ng total RNA using PrimeScript RTase (TaKaRa Biotechnology, Dalian, China). The concentration of the cDNA was adjusted using PCR and the grape *Actin1* gene (GenBank accession number AY680701) with the primers F (5′-GATTCTGGTGATGGTGTGAGT-3′) and R (5′-GACAATTTCCCGTTCAGCAGT-3′). Gene-specific primers for each *VvWRKY* gene were designed using Primer Premier 5.0 and optimized using oligo 7 (Supplementary Table S1 available at *JXB* online). Semiquantitative reverse-transcription (RT) PCR reactions were conducted using the following profile: initial denaturation at 94°C for 2min, followed by 30–40 cycles of denaturation at 92°C for 30 s, annealing at 60±5°C for 30 s, extension at 72°C for 30 s, and final extension at 72°C for 2min. PCR products were separated on a 1.5% (w/v) agarose gel with ethidium bromide staining and imaged under UV light for further gene expression analysis. Each reaction was repeated three times and the three independent analyses showed the same trends for each gene and treatment. The expression data from the semiquantitative RT-PCR were collated, analysed, and visualized using the programs GeneSnap and Mev 4.8.1 (Saeed *et al.*, 2006).

### Real-time quantitative PCR

Real-time quantitative PCR was conducted using SYBR green (TaKaRa Biotechnology) on an IQ5 real time-PCR machine (Bio-Rad, Hercules, CA, USA) with a final volume of 20 μl per reaction. Each reaction mixture contained 10.0 μl SYBR Premix Ex Taq II (TaKaRa Biotechnology), 1.0 μl cDNA template, 0.8 μl each primer (1.0 μM), and 7.4 μl sterile distilled H_2_O. Each reaction was performed in triplicate. Cycling parameters were 95°C for 30 s, 40 cycles at 95°C for 5 s, and 60°C for 30 s. Melt-curve analyses were performed using a program with 95°C for 15 s and then a constant increase from 60°C to 95°C. Gene-specific DNA primers were the same as those used for semiquantitative RT-PCR (Supplementary Table S1). The grape *Actin1* gene was used as the internal reference gene. The software IQ5 was used to analyse the relative expression levels using the normalized-expression method (Hou *et al.*, 2013).

## Results

### Identification of *WRKY* genes in the grape (*V. vinifera* L.) genome

A total of 61 genes were originally obtained between PF03106 (a Hidden Markov Model profile of WRKY DNA-binding domain) and Grape Genome (12X) with BLASTP. Based on the presence of apparently complete WRKY domains, 59 genes were subsequently selected and annotated as being grape *WRKY* genes. Genes without a complete predicted WRKY domain were removed (*GSVIVT01016690001*, *GSVIVT01031401001*). Further analysis of the protein sequences using the NCBI website revealed that GSVIVT01016690001 shares 63% similarity with a *Theobroma cacao* GDSL-motif lipase protein, indicating that GSVEVT01016690001 may not belong to the WRKY family. The other putative 59 grape *WRKY* genes were mapped onto the 19 grape chromosomes and then renamed from *VvWRKY1* to *VvWRKY59* based on their distributions and relative linear orders among the respective chromosome. *VvWRKY4* (GSVIVT01001332001) was located to chromosome 1_random region and *VvWRKY59* (GSVIVT01007006001) in an unknown region, and were thus unlike the other 57 *VvWRKY* genes. At least one EST for 52 *VvWRKY* genes was found in the public *V. vinifera* EST database at NCBI and used for further corroboration, while only seven (*VvWRKY9*, *VvWRKY13*, *VvWRKY 14*, *VvWRKY 17*, *VvWRKY 24*, *VvWRKY 41*, *VvWRKY 51*) were without a corresponding EST. However, in order to perform a systematic and comprehensive analysis of all of the *VvWRKY* genes, 59 specific primers were designed (Supplementary Table S1) for the expression analyses and gene confirmation. Detailed information about each *VvWRKY* gene is showed in Table 1, including the *WRKY* gene group numbers, gene locus numbers, accession numbers for the full-length sequences at NCBI, chromosome distribution (start sites and end sites), and the length of coding sequences.

**Table 1.** Grape *WRKY* genes and accessionsCDS, coding sequence; NG, no group; NSG, no subgroup, ORF, open reading frame.

Group	Subgroup	Gene ID	Gene locus ID	Accession no.	CDS (bp)	ORF (aa)	Chromosome	Start site	End site	Full length
I		*VvWRKY4*	GSVIVT01001332001	CBI36506.3	1308	435	1_random	297 660	312 015	14 356
		*VvWRKY59*	GSVIVT01007006001	CBI33229.3	1653	550	Unknown	29 694 308	29 699 639	5332
		*VvWRKY46*	GSVIVT01011472001	CBI22264.3	2670	889	14	29 916 400	29 925 511	9112
		*VvWRKY58*	GSVIVT01014854001	CBI39865.3	1869	622	19	10 665 036	10 669 055	4020
		*VvWRKY15*	GSVIVT01019109001	CBI17638.3	1461	486	4	16 664 476	16 666 741	2266
		*VvWRKY35*	GSVIVT01023600001	CBI36524.3	1500	499	11	7 835 815	7 846 137	10 323
		*VvWRKY18*	GSVIVT01024624001	CBI15865.3	1713	570	6	8 290 025	8 295 109	5085
		*VvWRKY28*	GSVIVT01025562001	CBI32633.3	1317	438	8	14 033 451	14 039 562	6112
		*VvWRKY39*	GSVIVT01030046001	CBI28412.3	1095	364	12	9 116 731	9 122 807	6077
		*VvWRKY26*	GSVIVT01030258001	CBI18092.3	1542	513	8	9 796 056	9 798 907	2852
		*VvWRKY11*	GSVIVT01035965001	CBI21139.3	1593	530	4	6 569 931	6 576 637	6707
		*VvWRKY57*	GSVIVT01037775001	CBI26736.3	1659	552	19	7 760 186	7 767 468	7283
II	a	*VvWRKY30*	GSVIVT01015952001	CBI25166.3	837	278	9	16 094 219	16 096 253	2035
	a	*VvWRKY9*	GSVIVT01035884001	CBI21068.3	789	262	4	5 247 592	5 248 886	1295
	a	*VvWRKY10*	GSVIVT01035885001	CBI21069.3	861	286	4	5 265 806	5 268 041	2236
	b	*VvWRKY54*	GSVIVT01008046001	CBI15258.3	1818	605	17	6 316 168	6 320 317	4150
	b	*VvWRKY45*	GSVIVT01011356001	CBI22167.3	1419	502	14	28 923 786	28 926 499	2714
	b	*VvWRKY31*	GSVIVT01012682001	CBI23209.3	1533	510	10	618 603	621 252	2650
	b	*VvWRKY2*	GSVIVT01020060001	CBI32009.3	1785	594	1	10 977 206	10 982 423	5218
	b	*VvWRKY22*	GSVIVT01028244001	CBI37053.3	1440	479	7	4 899 927	4 903 175	3249
	b	*VvWRKY40*	GSVIVT01029688001	CBI19998.3	1473	490	12	13 065 135	13 099 628	34 494
	b	*VvWRKY38*	GSVIVT01030453001	CBI28821.3	1497	498	12	5 678 707	5 681 171	2465
	b	*VvWRKY56*	GSVIVT01037686001	CBI26664.3	1491	496	19	6 882 419	6 884 988	2570
	c	*VvWRKY53*	GSVIVT01008553001	CBI15677.3	456	151	17	922 600	925 171	2572
	c	*VvWRKY3*	GSVIVT01010525001	CBI27681.3	570	189	1	21 460 123	21 461 397	1275
	c	*VvWRKY1*	GSVIVT01012196001	CBI27268.3	852	283	1	628 682	633 595	4914
	c	*VvWRKY47*	GSVIVT01018300001	CBI16682.3	687	228	15	11 498 970	11 504 729	5760
	c	*VvWRKY37*	GSVIVT01020864001	CBI22108.3	936	311	12	878 934	881 289	2356
	c	*VvWRKY33*	GSVIVT01021397001	CBI30827.3	960	319	10	4 894 476	4 896 340	1865
	c	*VvWRKY24*	GSVIVT01022245001	CBI21522.3	582	193	7	17 794 379	17 797 240	2862
	c	*VvWRKY25*	GSVIVT01022259001	CBI21534.3	681	226	7	17 958 306	17 960 930	2625
	c	*VvWRKY49*	GSVIVT01026969001	CBI40411.3	606	201	15	18 940 954	18 942 146	1193
	c	*VvWRKY21*	GSVIVT01028147001	CBI36970.3	909	302	7	4 200 160	4 202 241	2082
	c	*VvWRKY44*	GSVIVT01033063001	CBI21329.3	549	182	14	25 479 103	25 481 683	2581
	c	*VvWRKY13*	GSVIVT01033194001	CBI24019.3	471	156	4	9 399 944	9 400 803	860
	c	*VvWRKY14*	GSVIVT01033195001	CBI24020.3	306	101	4	9 409 805	9 411 286	1482
	c	*VvWRKY16*	GSVIVT01034968001	CBI22862.3	930	309	5	530 084	531 773	1690
	c	*VvWRKY8*	GSVIVT01035426001	CBI20684.3	501	166	4	1 209 585	1 211 712	2128
	d	*VvWRKY19*	GSVIVT01000752001	CBI17951.3	855	284	7	381 035	383 836	2802
	d	*VvWRKY7*	GSVIVT01001286001	CBI31897.3	318	105	2	4 989 461	4 989 778	318
	d	*VvWRKY55*	GSVIVT01009441001	CBI19480.3	960	319	18	8 391 930	8 393 726	1797
	d	*VvWRKY23*	GSVIVT01022067001	CBI21376.3	843	280	7	16 322 549	16 324 116	1568
	d	*VvWRKY36*	GSVIVT01029265001	CBI17876.3	840	279	11	17 821 900	17 823 266	1367
	d	*VvWRKY12*	GSVIVT01033188001	CBI24014.3	804	267	4	9 363 169	9 365 026	1858
	d	*VvWRKY43*	GSVIVT01036223001	CBI35175.3	915	304	14	8 753 538	8 756 300	2763
	e	*VvWRKY32*	GSVIVT01021252001	CBI30716.3	837	278	10	3 008 687	3 010 451	1765
	e	*VvWRKY5*	GSVIVT01019419001	CBI34393.3	972	323	2	512 163	513 533	1371
	e	*VvWRKY34*	GSVIVT01021765001	CBI31090.3	1266	421	10	10 755 760	10 759 820	4061
	e	*VvWRKY50*	GSVIVT01026965001	CBI40407.3	1047	348	15	18 957 231	18 958 817	1587
	e	*VvWRKY20*	GSVIVT01028129001	CBI36956.3	729	242	7	4 044 128	4 045 807	1680
	e	*VvWRKY51*	GSVIVT01028823001	CBI22627.3	549	182	16	18 360 079	18 360 711	633
	NSG	*VvWRKY29*	GSVIVT01034148001	CBI30536.3	900	299	8	14 828 040	14 830 056	2017
	NG	*VvWRKY17*	GSVIVT01025491001	CBI16558.3	366	121	6	364 396	365 387	992
III		*VvWRKY6*	GSVIVT01019511001	CBI34466.3	1029	342	2	1 228 314	1 229 702	1389
		*VvWRKY48*	GSVIVT01027069001	CBI40493.3	1083	360	15	18 191 021	18 193 489	2469
		*VvWRKY52*	GSVIVT01028718001	CBI22547.3	1095	364	16	19 477 141	19 479 868	2728
		*VvWRKY27*	GSVIVT01030174001	CBI18028.3	996	331	8	10 843 756	10 846 082	2327
		*VvWRKY42*	GSVIVT01032661001	CBI25345.3	867	288	13	1 719 393	1 720 884	1492
		*VvWRKY41*	GSVIVT01032662001	CBI25346.3	927	308	13	1 716 836	1 718 836	2001

### Multiple sequence alignment of *VvWRKY* genes

**Fig. 1.**
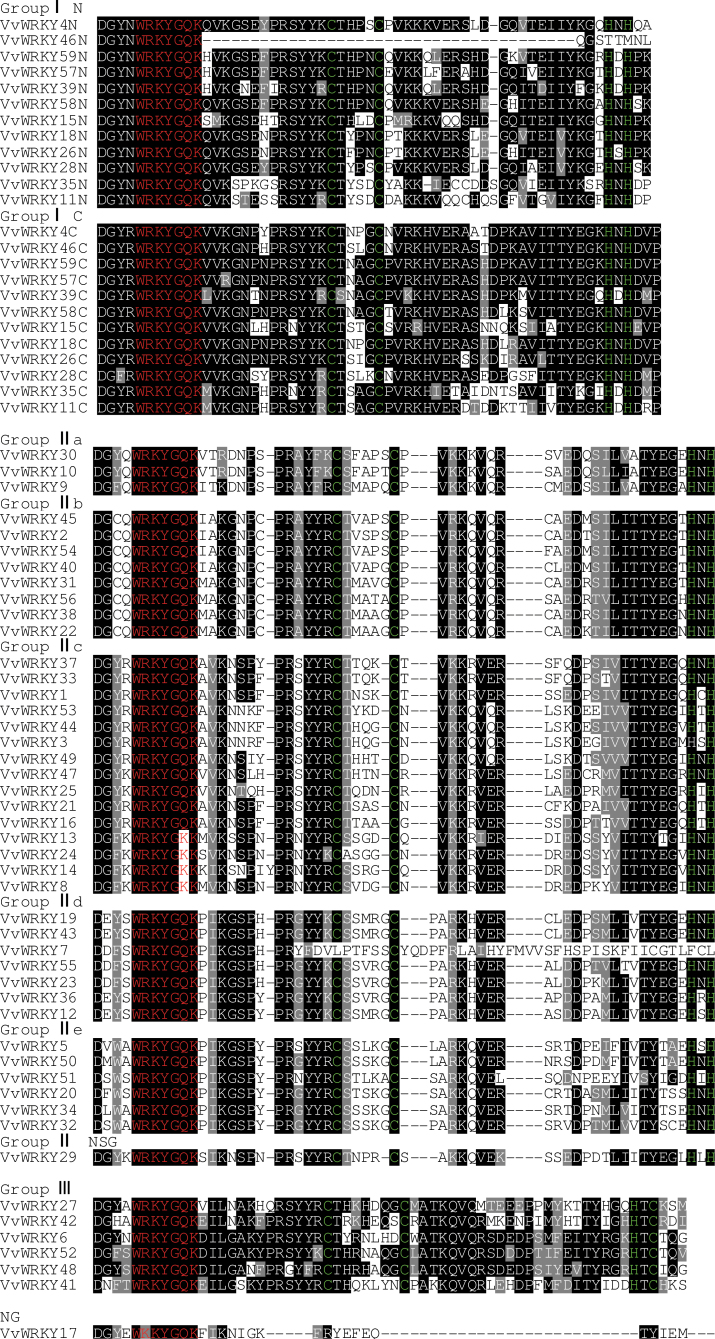
Multiple sequence alignment of the WRKY domain among *V. vinifera WRKY* genes. Red indicates conserved WRKY amino acid domains; green indicates zinc-finger motifs; dashes indicate gaps. ‘N’ and ‘C’ indicate the N-terminal and C-terminal WRKY domain of a specific *WRKY* gene (this figure is available in colour at *JXB* online).

The most prominent structural feature of WRKY proteins is the WRKY domain, which has been shown to interact with the W-box (C/T)TGAC(T/C), thereby activating a large number of defence-related genes (Eulgem *et al.*, 2000). The WRKY domain consists of a highly conserved hepta-peptide stretch of WRKYGQK at the N-terminus, followed by a zinc-finger motif (Eulgem *et al.*, 2000). A multiple sequence alignment of the core WRKY domain, spanning approximately 60 amino acids of all 59 *VvWRKY* proteins is shown in Fig. 1. A total of 54 *VvWRKY* proteins were found to have the highly conserved sequence WRKYGQK, while the others (*VvWRKY8*, *VvWRKY13*, *VvWRKY14*, *VvWRKY17*, *VvWRKY24*) vary by a single amino acid. Of these WRKYGKK is the most common, consistent with studies in tomato, being present in four of the five variants, while *VvWRKY17* contains a WKKYGQK sequence. One possible consequence of variation in this WRKY domain is an altered binding specificity in the DNA targets, but this remains to be demonstrated. As previously described (Eulgem *et al.*, 2000), the metal-chelating zinc-finger motif (C–X_4–5_–X_22–23_–H–X_1_–H or C–X_7_–C–X_23_–H–X_1_–C) is another important structural characteristic of WRKY proteins. However, some incomplete zinc-finger motifs were also identified, including examples encoded by *VvWRKY7*, *VvWRKY17*, and *VvWRKY46*. Interestingly, in contrast to group-III WRKY in rice (Wu *et al.*, 2005) and barley (Mangelsen *et al.*, 2008), there are no *VvWRKY* in group-III proteins containing a C–X_7_–C–X_24_–H–X–C zinc-finger motif, perhaps suggesting that this is a feature of monocotyledonous species.

### Phylogenetic analysis of *WRKY* genes from grape, *Arabidopsis*, and tomato

**Fig. 2.**
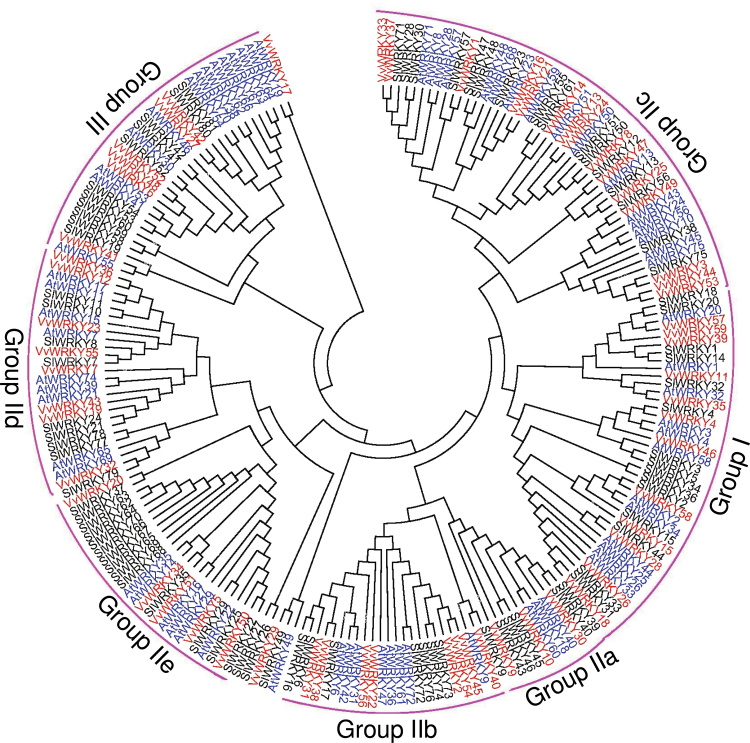
Phylogenetic tree of *WRKY* genes among grape (red), *Arabidopsis* (blue), and tomato (black). Filled circle lines are used to cluster the genes with similar structures and functions (this figure is available in colour at *JXB* online).

To further analyse the evolutionary relationships in the *VvWRKY* gene family and to help in their classification, a total of 210 *WRKY* genes, comprising 70 from *Arabidopsis*, 81 from tomato, and 59 from grape, were used to construct a phylogenetic tree (Fig. 2). Based on the number of WRKY domains and the features of the specific zinc-finger motifs, all 59 *VvWRKY* genes were classified into three main groups, with five subgroups in group II (Eulgem *et al.*, 2000). Twelve grape *WRKY* genes (*VvWRKY4*, *VvWRKY11*, *VvWRKY15*, *VvWRKY18*, *VvWRKY26*, *VvWRKY28*, *VvWRKY35*, *VvWRKY39*, *VvWRKY46*, *VvWRKY57*, *VvWRKY58*, *VvWRKY59*) with two WRKY domains belong to group I, which have a zinc-finger motif of C–X_4_–C–X_22–23_–H–X_1_–H. The other 40 grape *WRKY* genes with the zinc-finger structure of C–X_4_–C–X_22–23_–H–X_1_–H were assigned to group II, which comprised 60% of the total number of *VvWRKY* genes. The 40 group-II *VvWRKY* genes are unevenly distributed amongst the five subgroups: group IIa (three: *VvWRKY9*, *VvWRKY10*, *VvWRKY30*), group IIb (eight: *VvWRKY2*, *VvWRKY22*, *VvWRKY31*, *VvWRKY38*, *VvWRKY40*, *VvWRKY45*, *VvWRKY54*, *VvWRKY56*), group IIc (15: *VvWRKY1*, *VvWRKY3*, *VvWRKY8*, *VvWRKY13*, *VvWRKY14*, *VvWRKY16*, *VvWRKY21*, *VvWRKY24*, *VvWRKY25*, *VvWRKY33*, *VvWRKY37*, *VvWRKY44*, *VvWRKY47*, *VvWRKY49*, *VvWRKY53*), group IId (seven: *VvWKY7*, *VvWRKY12*, *VvWRKY19*, *VvWRKY23*, *VvWRKY36*, *VvWRKY43*, *VvWRKY5*5), and group IIe (six: *VvWRKY5*, *VvWRKY20*, *VvWRKY32*, *VvWRKY34*, *VvWRKY 50*, *VvWRKY51*). In contrast to group I, group-II genes have only one WRKY domain. Instead of the C_2_H_2_ pattern, group-III genes contain a C_2_HC zinc-finger motif (C–X_7_–C–X_23_–H–X_1_–C) and six of the 59 *VvWRKY* genes (*VvWRKY6*, *VvWRKY27*, *VvWRKY41*, *VvWRKY42*, *VvWRKY48*, *VvWRKY52*) belong to this group. Finally, *VvWRKY17* and *VvWRKY29* do not group within any of the other group-II subgroups, possibly because of their apparently incomplete structures. Detailed information about the classification of the genes and the WRKY domains, as well as the profile of zinc-finger motifs can be found in Table 1 and Supplementary Table S2, respectively.

### Exon–intron organization of *VvWRKY* genes

**Fig. 3.**
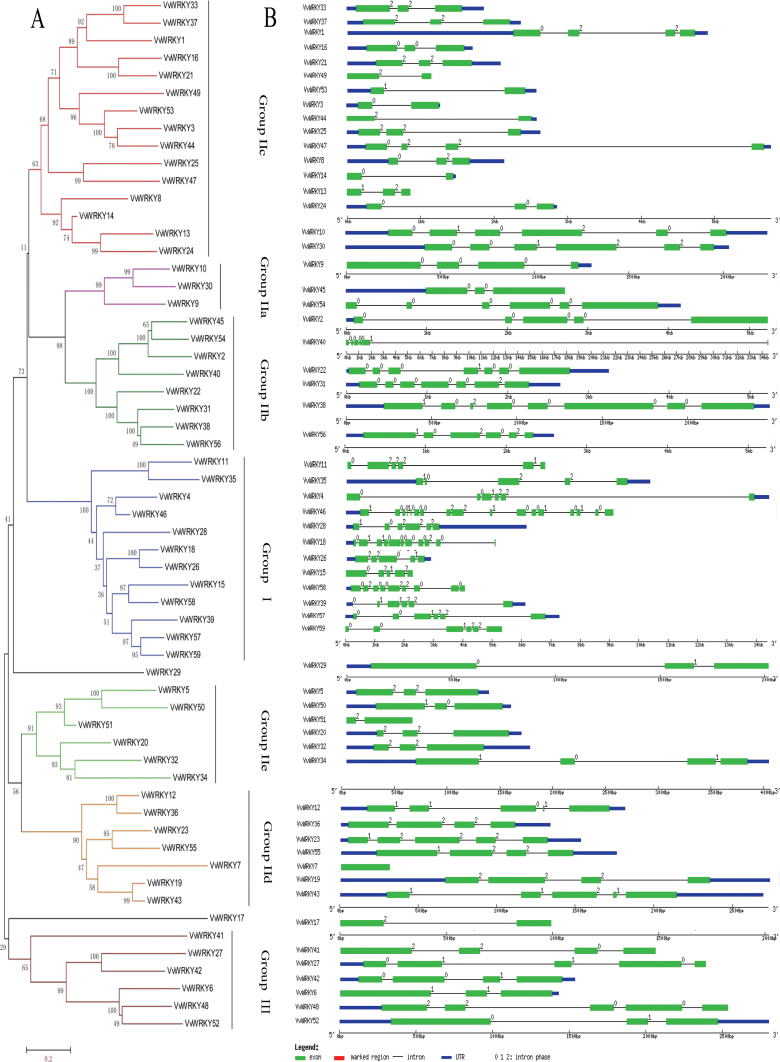
Genomic organization of grape *WRKY* genes. (A) Unrooted phylogenetic tree built on the basis of 59 complete WRKY-domain proteins in grape; details of clusters are shown in different colours. (B) Exon–intron structure of grape *WRKY* genes; blue indicates untranslated 5′- and 3′-regions; green indicates exons; black indicates introns (this figure is available in colour at *JXB* online).

Insights into the structures of the *VvWRKY* genes were obtained through an analysis of the exon/intron boundaries, which are known to play important roles in the evolution of multiple gene families (Zhang *et al.*, 2012). As shown in Fig. 3, 52 of the 59 *VvWRKY* genes have two–six exons (seven with two exons, 16 with three exons, nine with four exons, nine with five exons, and 11 with six exons). The fact that *VvWRKY46* has 17 exons, *VvWRKY18* has 11 exons, *VvWRKY58* has nine exons, *VvWRKY38* has eight exons, *VvWRKY22* and *VvWRKY31* have seven exons and *VvWRKY7* has only one exon indicates that both exon loss and gain has occurred during the evolution of the *WRKY* gene family. This may lead to functional diversity of closely related *WRKY* genes; however, this study noted that *VvWRKY* genes in the same group usually have a similar number of exons. The number of exons in group I is relatively large, ranging from five to 11 (*VvWRKY46* with 17 exons was not included because of the possibility of special variation), while genes in group II have a relatively small number, ranging from one to eight exons, and group III from three to five. This exon pattern similarity may be the consequence of a number of gene duplication events. A further analysis indicated that nearly all *VvWRKY* genes contained an intron in their respective WRKY domains (Wu *et al.*, 2005; He *et al.*, 2012). The R-type introns are widely exist in majority of WRKY groups (I, IIc, IId, IIe, III), the same cases in rice and *Arabidopsis* (Eulgem *et al.*, 2000; Wu *et al.*, 2005).

### Tandem duplication of *VvWRKY* genes

**Fig. 4.**
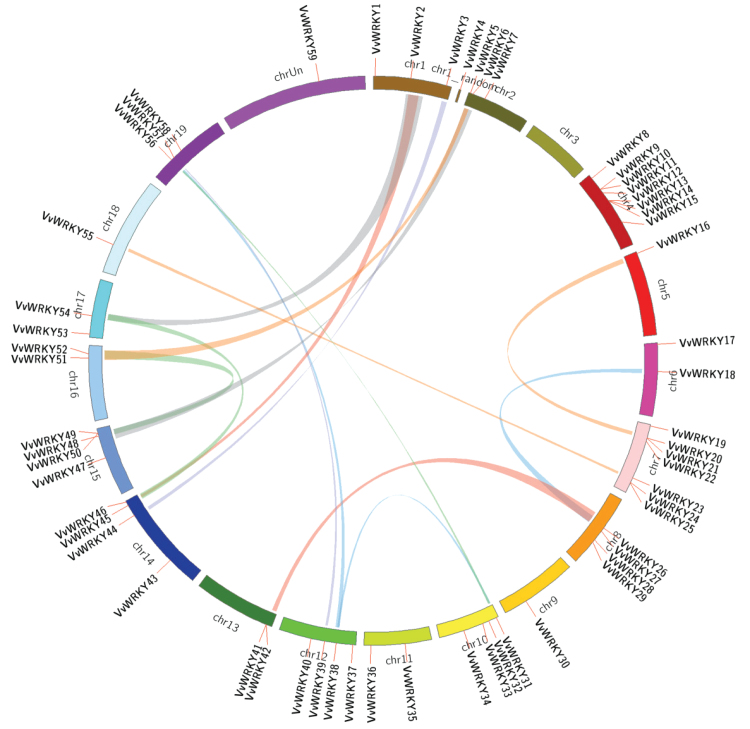
Chromosome distribution and synteny analysis of grape *WRKY* genes. Chromosomes 1–19 are shown with different colours and in a circular form. The approximate distribution of each *VvWRKY* gene is marked with a short red line on the circle. Coloured curves denote the details of syntenic regions between grape *WRKY* genes (this figure is available in colour at *JXB* online).

Genomic comparison is a method for rapidly transferring available genomic information from a model species to a less-studied species (Lyons *et al.*, 2008; Zhang *et al.*, 2012). The current work analysed the tandem duplication events of the 59 *VvWRKY* genes on the 19 grape chromosomes (Table 3) according to the methods of Holub (2001), where a chromosomal region within 200kb containing two or more genes is defined as a tandem duplication event. There were 13 *VvWRKY* genes (*VvWRKY9*, *VvWRKY10*, *VvWRKY12*, *VvWRKY13*, *VvWRKY14*, *VvWRKY20*, *VvWRKY21*, *VvWRKY24*, *VvWRKY25*, *VvWRKY41*, *VvWRKY42*, *VvWRKY49*, *VvWRKY50*) clustered into six tandem duplication event regions on grape chromosome 4 (two clusters), 7 (two clusters), 13 (one cluster) and 15 (one cluster) (Table 3). Chromosomes 4 (cluster 1 and cluster 2) and 7 (cluster 3 and cluster 4) had two clusters respectively, indicating a hot spot of *WRKY* gene distribution. Besides the tandem duplication events, 15 segregation duplication events were also identified (Fig. 4 and Supplementary Table S3), indicating that some *VvWRKY* genes were possibly generated by gene duplication. Moreover, the segregation duplication events can also provide a reference for the *WRKY* gene evolutionary relationship and functional prediction.

### Synteny analysis of *VvWRKY* genes

**Fig. 5.**
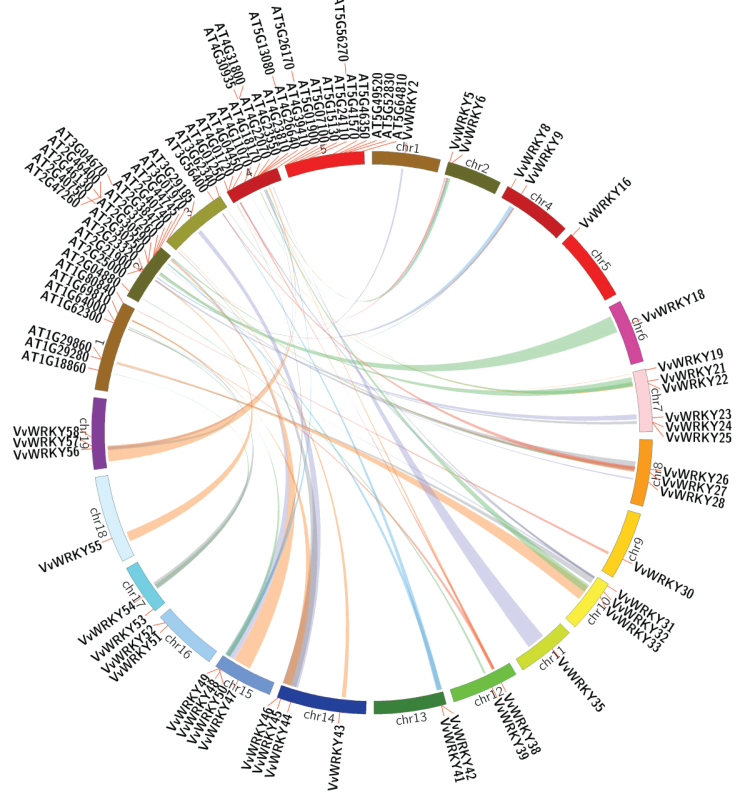
Synteny analysis of grape and *Arabidopsis WRKY* genes. The chromosomes of grape and *Arabidopsis* are depicted as a circle. The approximate distribution of each *AtWRKY* gene and *VvWRKY* gene is marked with a short red line on the circle. Coloured curves denote the details of syntenic regions between grape and *Arabidopsis WRKY* genes (this figure is available in colour at *JXB* online).

A substantial number of *WRKY* genes from the model plant *Arabidopsis* have been systematically investigated (Eulgem *et al.*, 2000; Dong *et al.*, 2003) and so the current work performed a synteny analysis of *Arabidopsis* and grape *WRKY* genes (Fig. 5) to determine whether this might provide some functional insights. A total of 66 pairs of syntenic relations were identified, including 50 *AtWRKY* genes and 39 *VvWRKY* genes. Three *AtWRKY* genes (*AtWRKY6*, *AtWRKY31*, *AtWRKY36*) and nine *VvWRKY* genes (*VvWRKY6*, *VvWRKY9*, *VvWRKY21*, *VvWRKY27*, *VvWRKY31*, *VvWRKY33*, *VvWRKY38*, *VvWRKY49*, *VvWRKY54*) were found to be associated with at least three synteny events and interestingly, of these 12 genes, six were in group IIb, including three *AtWRKY* genes and three *VvWRKY* genes (*VvWRKY31*, *VvWRKY38*, *VvWRKY54*). This may indicate a high conservation of group-IIb *WRKY* genes, which may in turn suggest a fundamental function in plant development. Detailed results from the comparative analysis are shown in Supplementary Table S4. The large number of synteny events suggests that many *WRKY* genes arose before the divergence of the *Arabidopsis* and grape lineages.

### Expression patterns of *VvWRKY* genes in different tissues

**Fig. 6.**
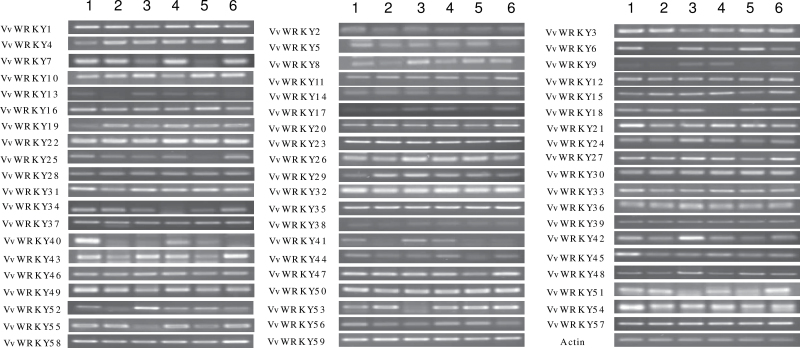
Expression pattern of 59 *VvWRKY* genes in organs/tissues of ‘Kyoho’ (*V. labrusca* × *V. vinifera*); *Actin1* (GenBank accession number AY680701) was used as an internal control. Lanes: 1: root, 2: tendril, 3: leaf, 4: inflorescence, 5: fruit, 6: stem. The experiments were repeated three times and the results were consistent.

To assess the potential functions of *VvWRKY* genes during grape development, this study investigated the expression patterns of all 59 *VvWRKY* genes in six organs/tissues (roots, tendrils, leaves, inflorescences, fruit, and stems). Nearly half of the *VvWRKY* genes showed no significant organ/tissue related differences in expression, but some clear spatial differences were noted (Fig. 6). For example, *VvWRKY40* and *VvWRKY45* were expressed at high levels in roots and *VvWRKY42* and *VvWRKY52* expression were particularly high in leaves. No *VvWRKY* gene was specifically associated with inflorescences or fruit. The *WRKY* genes which showed no significant expression difference between tissues are likely to play a more ubiquitous role in grape.

### Expression profiles of *VvWRKY* genes in response to drought, salt, and powdery mildew infection

**Fig. 7.**
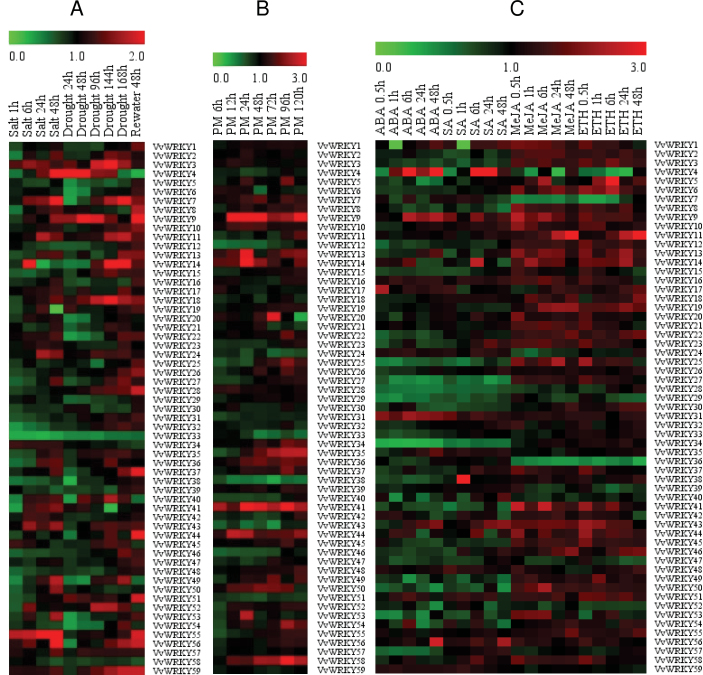
Expression profiles of 59 *VvWRKY* genes. The results of semiquantitative RT-PCR were quantified using the Gene Tools software, and the relative expression levels of *VvWRKY* genes under treatments compared to the controls were used for hierarchical cluster analysis with MeV 4.8.1. The colour scale represents relative expression levels, with red as increased transcript abundance and green as decreased transcript abundance (A) Expression profiles of *VvWRKY* genes under biotic stress treatments, salinity, and drought (original results shown in Supplementary Figs S1 and S2). (B) Expression profiles of *VvWRKY* genes under biotic stress treatment, powdery mildew (original results shown in Supplementary Fig. S3). (C) Expression profiles of *VvWRKY* genes following four hormone treatments (abscisic acid (ABA), salicylic acid (SA), methyl jasmonic acid (MeJA), and ethanol (Eth)) (original results shown in Supplementary Figs S4–S7). The experiments were repeated three times and the results were consistent (this figure is available in colour at *JXB* online).

**Fig. 8.**
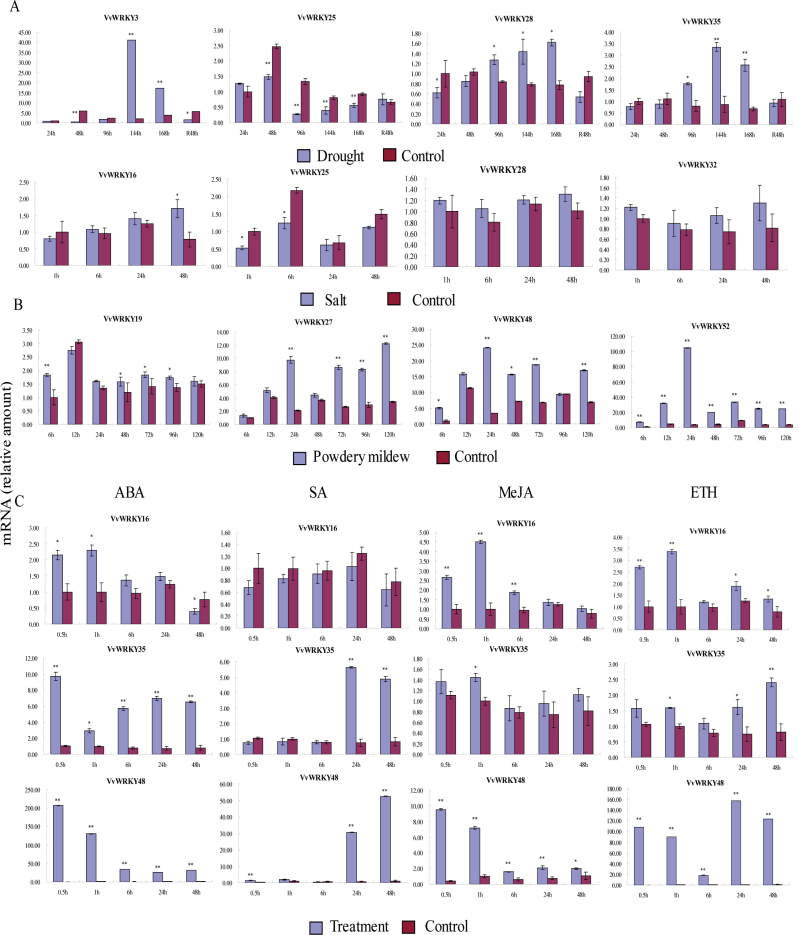
Real-time quantitative PCR expression levels of selected *VvWRKY* genes following salinity stress, drought stress treatments, powdery mildew inoculation, and various hormone treatments (abscisic acid (ABA), salicylic acid (SA), methyl jasmonic acid (MeJA), and ethanol (Eth)). The expression levels were normalized to 1h (salt stress treatment), 24h (drought stress treatment), 6h (powdery mildew inoculation), and 0.5h (hormone treatments) CK sample, respectively. Mean values and SDs were obtained from three biological and three technical replicates. Asterisks indicate the corresponding gene significantly up- or downregulated under the differential treatment by t-test (*P<0.05, **P<0.01). (A) Expression levels of selected *VvWRKY* genes under abiotic stresses (salt and drought). (B) Expression levels of selected *VvWRKY* gene under biotic stress (powdery mildew). (C) Expression levels of selected *VvWRKY* genes under hormone treatments (this figure is available in colour at *JXB* online).

The ability of plants to tolerate a variety of abiotic and biotic stresses is an essential adaptive feature in changing environments. Moreover, the identification and functional analysis of genes involved in biotic and abiotic stress signal transduction pathways is of considerable interest in the context of enhancing agricultural productivity. In order to obtain insights into the potential roles of all 59 *VvWRKY* genes in stress associated gene expression, this study used 2-year-old ‘Kyoho’ (*V. labrusca* × *V. vinifera*) seedlings growing in pots in the greenhouse of Northwest A&F University that were exposed to salt and drought stress and monitored expression using semiquantitative RT-PCR, ‘Shang-24’ was used for powdery mildew inoculation. A total of four *VvWRKY* genes that could be associated with salt stress (*VvWRKY16*, *VvWRKY25*, *VvWRKY28*, *VvWRKY35*), drought stress (*VvWRKY3*, *VvWRKY25*, *VvWRKY28*, *VvWRKY35*), and powdery mildew inoculation (*VvWRKY19*, *VvWRKY27*, *VvWRKY48*, *VvWRKY52*), based on semiquantitative RT-PCR analysis (Fig. 7A and B and Supplementary Figs S1–S3) were selected for further analysis and validation using real-time quantitative PCR (Fig. 8A and B). In most cases a similar result was seen, including the four genes that were induced by drought stress and powdery mildew inoculation and three genes (*VvWRKY16*, *VvWRKY25*, *VvWRKY35*) by salt stress treatment. Only one gene (*VvWRKY28*) showed no significant difference in the real-time quantitative PCR.

Based on the semiquantitative RT-PCR data (Fig. 7A), *VvWRKY* genes tend to be downregulated to a greater degree by salinity stress than by drought stress. *VvWRKY12*, *VvWRKY14*, *VvWRKY15*, *VvWRKY26*, *VvWRKY28*, *VvWRKY31*, *VvWRKY32*, *VvWRKY39*, *VvWRKY46*, and *VvWRKY48*, which all showed clear downregulation by the salt stress treatment, were upregulated to varying degrees by the drought stress treatment, indicating distinctly different regulatory networks existence. The reaction time for the expression pattern changes was another focus of this study and some genes, such as *VvWRKY1* and *VvWRKY51*, showed altered expression at an early time point (1h under salt stress, 24h under drought stress), while others, including *VvWRKY57* and *VvWRKY59* showed upregulated expression at a relatively late time (48h under salt stress, 144 or 168h under drought stress). On the other hand, some genes showed an early downregulation but subsequent upregulation (*VvWRKY2*, *VvWRKY27*, *VvWRKY49*): for example, *VvWRKY2* was downregulated at 1h after the onset of the salt stress treatment, but was upregulated after 48h for example.

The association of the *VvWRKY* genes with biotic stress was investigated using infection with powdery mildew, a severe disease worldwide (Zhang *et al.*, 2012). The semiquantitative RT-PCR based expression profiles (Fig. 7B) of *VvWRKY4*, *VvWRKY11*, *VvWRKY12*, *VvWRKY23*, *VvWRKY24*, *VvWRKY30*, *VvWRKY31*, *VvWRKY32*, *VvWRKY33*, *VvWRKY38*, *VvWRKY40*, *VvWRKY42*, *VvWRKY43*, *VvWRKY46*, *VvWRKY47*, and *VvWRKY51* indicated a potentially negative effect on grape powdery mildew resistance, while *VvWRKY7*, *VvWRKY8*, *VvWRKY9*, *VvWRKY13*, *VvWRKY14*, *VvWRKY25*, *VvWRKY26*, *VvWRKY27*, *VvWRKY34*, *VvWRKY35*, *VvWRKY36*, *VvWRKY37*, *VvWRKY39*, *VvWRKY41*, *VvWRKY44*, *VvWRKY45*, *VvWRKY48*, *VvWRKY49*, *VvWRKY50*, *VvWRKY52*, *VvWRKY55*, and *VvWRKY58* showed the opposite pattern. *VvWRKY12*, *VvWRKY23*, *VvWRKY40 VvWRKY43*, and *VvWRKY46* showed significant downregulation during early infection (6–72h or to 96h), while *VvWRKY34*, *VvWRKY35*, and *VvWRKY36* were upregulated during a later stage (24–120h), possibly indicating a relationship between expression pattern and responding time.

### Expression profiles of *VvWRKY* genes to hormone treatments

Plant hormones such as ABA, SA, MeJA, and ethylene have well-established roles in modulating plant signalling networks (Fujita *et al.*, 2006). In this study, hormone treatments resulted in a wide variety of *VvWRKY* gene expression profiles (Fig. 7C and Supplementary Figs S4–S7). A total of 31 *VvWRKY* genes showed different degrees of downregulation by the ABA treatment while 14 were upregulated. Similarly, 19 *VvWRKY* genes were downregulated and 18 were upregulated expression following SA treatment. However, the expression profiles resulting from MeJA and Eth treatments were distinct from those modulated by ABA and SA and a substantially greater number of upregulated genes were observed: 37 were upregulated by MeJA and 11 were downregulated, while 37 were upregulated and six were downregulated by Eth treatment. These expression variations indicate that the *VvWRKY* gene family possible is collectively regulated by a broad set of hormonal signals.

## Discussion

Many *WRKY* family genes play important roles in diverse plant developmental and physiological processes (Du and Chen, 2000; Eulgem *et al.*, 2000), including embryogenesis (Lagace and Matton, 2004), seed coat and trichome development (Johnson *et al.*, 2002), and leaf senescence (Miao and Zhetgraf, 2007; Zhou *et al.*, 2011), as well as various plant abiotic and biotic stress responses. This current study describes the identification of 59 *VvWRKY* genes from grape, together with an analysis of their structure, evolutionary history, and expression pattern diversity with respect to biotic and abiotic stresses.

### Identification and annotation of *VvWRKY* genes

The publicly available collection of *V. vinifera* ESTs were used to confirm the identity of the 59 *VvWRKY* genes that were initially identified amongst the 30 434 annotated grape genes using BLASTP. Of the 59 *VvWRKY* genes, seven were without supporting EST data, indicating that they may not be expressed during grape development. However, semiquantitative RT-PCR analysis was used to confirm that all 59 *VvWRKY* genes, including the seven genes without EST support, were indeed expressed and had putative functions in various aspects of grape biology, indicating all these 59 *VvWRKY* genes were putative *WRKY* genes in grape.

### Structural conservation and divergence of *VvWRKY* genes

Comparative genomic analysis is an effective method for studying gene structures and so this study assessed the conserved structural domains of the predicted grape WRKY proteins. Multiple sequence alignments revealed that five *VvWRKY* proteins (*VvWRKY8*, *VvWRKY13*, *VvWRKY14*, *VvWRKY17*, *VvWRKY24*) had sequence variations in their WRKY domain and three (*VvWRKY7*, *VvWRKY17*, *VvWRKY46*) had variations in their zinc-finger motif. In previous studies of *Arabidopsis AtWRKY* transcription factors, it was found that the binding-site preferences of the WRKYGQK motif depend on the DNA sequences adjacent to the TTGACY core motif (Ciolkowski *et al.*, 2008). In the genes recognized by the proteins with a variation to the WRKY domain, the TTGACY core motif may exhibit an altered structure and function, and so *WRKY* genes without the WRKYGQK motif may recognize binding sequences other than the W-box element ((C/T)TGAC(C/T)). In tobacco, the NtWRKY12 protein, which contains a WRKYGKK motif, recognizes the downstream binding sequence TTTTCCAC, which is substantially different from the W-box (van Verk *et al.*, 2008). Moreover, the soybean WRKY proteins *GmWRKY6* and *GmWRKY21*, which have a WRKYGKK motif, do not bind normally to the W-box (Zhou *et al.*, 2008). It therefore seems that variations in the WRKYGQK motif influence the normal interaction of *WRKY* genes with downstream target genes, and so it would be interesting to investigate the functions and binding specificities of *VvWRKY8*, *VvWRKY13*, *VvWRKY14*, *VvWRKY17*, and *VvWRKY24*. Moreover, as far as is known, nothing is known about the effect of zinc-finger motif changes. It is possible that the variations of the WRKY domain and zinc-finger motif could influence the classification of the *VvWRKY* genes reported here. One example of this is *VvWRKY17*, which has a divergent zinc-finger sequence, leading to a nebulous classification in the phylogenetic tree. It remains to be determined whether the observed sequenced variations in the conserved domains affect the function or the expression patterns of the regulated gene targets.

Exon–intron structural diversification also plays an important role in the evolution of many gene families and exon–intron gain or loss may be caused by the rearrangement and fusions of different chromosome fragments (Xu *et al.*, 2012; Guo *et al.*, 2013). The current study provides an example of such diversification in the form of a *WRKY* gene (*VvWRKY7*) with only one exon, while other genes in the same phylogenetic group (group IIb) have four or five exons. Moreover, *VvWRKY46* has as many as 17 exons, while *VvWRKY4*, which is similar to *VvWRKY17*, only has six exons.

### The evolutionary relationship of *VvWRKY* genes

The size of the grape *WRKY* gene family (59) is small compared to that of other experimental model plants such as *Arabidopsis* (72) and rice (96). The *WRKY* genes from grape, *Arabidopsis*, rice, and tomato align within distinct phylogenetic groups (Table 2) and it is apparent that variations in the number of *WRKY* genes in group III are the primary cause of the diversity of *WRKY* gene family size. Interestingly, previous studies described group-III *WRKY* genes as being a newly defined group, as well as being the most dynamic group with respect to gene family evolution (Zhang and Wang, 2005). Therefore, a key role of group-III *WRKY* genes in plant evolution may exist.

**Table 2.** The number of *WRKY* genes in each phylogenetic group from *Arabidopsis*, rice, grape, and tomato*VvWRKY17*, *VvWRKY29*, *SlWRKY26*, *SlWRKY27*, and *SlWRKY49* are not included, since they could not be placed.

Gene	Phylogenetic group						
	I	IIa	IIb	IIc	IId	IIe	III
*AtWRKY*	13	4	7	18	7	9	14
*OsWRKY*	15	4	8	15	7	11	36
*VvWRKY*	12	3	8	15	7	6	6
*SlWRKY*	15	5	8	16	6	17	11

**Table 3.** Tandem duplication events in the 59 *VvWRKY* genes

Cluster number	Gene ID	Chromosome	Start site	End site
1	*VvWRKY9*	4	5 247 592	5 248 886
	*VvWRKY10*	4	5 265 806	5 268 041
2	*VvWRKY12*	4	9 363 169	9 365 026
	*VvWRKY13*	4	9 399 944	9 400 803
	*VvWRKY14*	4	9 409 805	9 411 286
3	*VvWRKY20*	7	4 044 128	4 045 807
	*VvWRKY21*	7	4 200 160	4 202 241
4	*VvWRKY24*	7	17 794 379	17 797 240
	*VvWRKY25*	7	17 958 306	17 960 930
5	*VvWRKY41*	13	1 716 836	1 718 836
	*VvWRKY42*	13	1 719 393	1 720 884
6	*VvWRKY49*	15	18 940 954	18 942 146
	*VvWRKY50*	15	18 957 231	18 957 231

Gene duplication events play a major role in genomic rearrangements and expansions (Vision *et al.*, 2000) and are defined as either tandem duplications, with two or more genes located on the same chromosome, or segmental duplications, with duplicated genes present on different chromosomes (Liu *et al.*, 2011). The large number of gene duplication events for grape (Figs 4 and 5) will help aid future gene function prediction and evolution analysis. Whole-genome duplication events (γ, β, α) are a common phenomenon in angiosperms (Zhang and Wang, 2005) and often lead to gene family expansion (Cannon *et al.*, 2004). Ling *et al.* (2011) reported that in cucumber (*Cucumis sativus*) *CsWRKY* family, a divergence generated in the number of group-III *WRKY* genes resulted from different style of duplication events that occurred after the divergence of the eurosids’ groups I and II (110 Mya). For some species in the eurosids’ group I (cucumber, soybean, and grape), the number of group-III *WRKY* genes is small, which may be caused by a different pattern of duplication events. This idea is consistent with the current results concerning the group-III *VvWRKY* genes. The group-III *AtWRKY* genes with normal tandem duplication events (*AtWRKY63*, *AtWRKY64*, *AtWRKY66*, *AtWRKY67*) show evidence of large-scale duplication. According to Zhang (2003) not all duplication events are stable, but instead can be fixed or lost in the population due to selection pressure and evolution. If the duplication events happens at a favourable time and in genes that are highly expressed, they will most likely be retained, but other duplication events that are not useful to the organism because of functional redundancy, or even a negative effect on plant development, will either be deleted from the genome or become very will diverge. The group-III *WRKY* gene divergence may reflect such a gene evolutionary selection.

### *VvWRKY* genes function in abiotic and biotic stresses

There is considerable evidence that *WRKY* genes play crucial roles in responses to abiotic and biotic stress-induced defence signalling pathways (Qiu *et al.*, 2004; Chen *et al.*, 2012; Ishihama and Yoshioka, 2012). From an applied perspective, the identification of *WRKY* genes with potential value in stress resistance improvement of grape would likely benefit from targeting such genes that are part of abiotic and biotic stress-response networks. The semiquantitative RT-PCR expression profiles generated in this study (Fig. 7) revealed different expression patterns (upregulation and downregulation) for each *VvWRKY* gene under specific treatments, thus providing a useful resource for future gene expression and functional analyses. Dong and coworkers (2003) showed that nearly 70% of the *AtWRKY* genes are differentially expressed in response to microbial infection or SA treatment. Consistent with these previous studies, the current results show that 70–90% of *VvWRKY* genes are differentially expressed following various abiotic and biotic stress treatments, highlighting the extensive involvement of *WRKY* genes in environmental adaptation.

The roles of many *Arabidopsis WRKY* genes in plant abiotic stress responses have been extensively studied recently. For example, *AtWRKY25* and *AtWRKY33* regulate plants adaptation to salinity stress through an interaction with their upstream or downstream target genes (Jiang and Deyholos, 2009). *VvWRKY26* shares 76% sequence similarity with *AtWRKY25* and 71% similarity with *AtWRKY33*, but in the current study, the change in expression profile of *VvWRKY26* as a consequence of the various treatments applied was not apparent. It is possible that although *VvWRKY26*, *AtWRKY25*, and *AtWRKY33* are segregation duplication genes, their function may differ in different plant species. The presence or absence of the regulatory elements in the duplicated genes can have an important consequence on subsequent divergence of gene function for an explanation.

*VvWRKY1* (Marchive *et al.*, 2007) and *VvWRKY2* (Mzid *et al.*, 2007) (named in this work *VvWRKY53* and *VvWRKY4*, respectively) have also been isolated and functionally analysed. *VvWRKY2* is known to activate the promoter of *VvC4H*, which is involved in the lignin biosynthesis pathway and cell-wall formation (Guillaumie *et al.*, 2010), and it likely plays an important role in resistance or tolerance to biotic and abiotic stress in plants. However, *VvWRKY4* appears not to respond to powdery mildew inoculation, suggesting that it may involve in resistance other abiotic and biotic stresses other than powdery mildew. On contrary, *VvWRKY53* is significantly upregulated at 24h post inoculation, which appears to share similar inoculation response with *VvWRKY1* as reported earlier (Marchive *et al.*, 2007), thus suggesting that *VvWRKY53* may play a role in eliciting resistance response during early stage of infection.

The temporal and spatial diversification of *WRKY* gene expression is widespread, which is important for gene function analysis. The *VvWRKY* genes *VvWRKY40*, *VvWRKY45*, *VvWRKY42*, and *VvWRKY52* had a higher expression in certain grape organs/tissues suggesting divergent roles for these genes in grape development. With regard to temporal diversification, this work considered the response time to the different treatments since previous studies had shown that some *WRKY* genes respond to drought stress at an early stage, such as *GsWRKY18*, which peaked at 0.5h after drought stress treatment (Ling *et al.*, 2011). The current data show the opposite pattern, since about half of the *VvWRKY* genes (*VvWRKY3* and *VvWRKY35*) selected for real-time quantitative PCR showed a peak of expression at 144h after treatment and one (*VvWRKY28*) peaked at 168h after drought stress treatment, indicating that a certain response time is needed for *VvWRKY* genes to respond to drought stress. The upregulation of the expression of some *WRKY* genes in response to powdery mildew inoculation showed a similar delay in response time, but shorter than that caused by the drought stress treatment (12h post treatment).

*WRKY* genes that are components of plant biotic stress regulatory networks have a complex response pattern. For example, *Arabidopsis AtWRKY33* (Lippok *et al.*, 2007) and *AtWRKY18* (Chen and Chen, 2002) can positively modulate defence-related gene expression and improve disease resistance, while some negative regulatory elements may prevent the overexpression of *AtWRKY33* and *AtWRKY18* and are detrimental to plant growth. Moreover, other *WRKY* genes, such as *AtWRKY7* (Kim *et al.*, 2006) and *AtWRKY48* (Xing *et al.*, 2008) have an immediate negative effect in the plant defence response. In these current studies, although *VvWRKY4*, *VvWRKY11*, *VvWRKY12*, *VvWRKY23*, *VvWRKY24*, *VvWRKY30*, *VvWRKY31*, *VvWRKY32*, *VvWRKY33*, *VvWRKY38*, *VvWRKY40*, *VvWRKY42*, *VvWRKY43*, *VvWRKY46*, *VvWRKY47*, and *VvWRKY51* may have a negative effect on grape powdery mildew resistance and *VvWRKY7*, *VvWRKY8*, *VvWRKY9*, *VvWRKY13*, *VvWRKY14*, *VvWRKY25*, *VvWRKY26*, *VvWRKY27*, *VvWRKY34*, *VvWRKY35*, *VvWRKY36*, *VvWRKY37*, *VvWRKY39*, *VvWRKY41*, *VvWRKY44*, *VvWRKY45*, *VvWRKY48*, *VvWRKY49*, *VvWRKY50*, *VvWRKY52*, *VvWRKY55*, and *VvWRKY58* may have a positive effect, the possible interactions between two or more genes and regulatory mechanisms remain to be resolved.

Phytohormones are critically important in coordinating regulatory networks and the signal transduction pathways associated with external cues. ABA is known to function in signalling in some stressful environments (Huang *et al.*, 2008), and SA, JA, and Eth play important roles in biotic stresses (Fujita *et al.*, 2006), while MeJA responds to some biotic stresses and wounding (Huang *et al.*, 2008). The current results show that the majority of *VvWRKY* genes showed significantly upregulated expression following treatment with MeJA and Eth, while ABA and SA treatments had the opposite effect. It has been reported that *AtWRKY33* can act as a positive regulator in Eth-mediated defence signalling against necrotrophic pathogens and as a negative regulator in SA-mediated responses for some biotrophic bacterial pathogens (Zheng *et al.*, 2006), which is the same trend seen in this study.

In conclusion, *WRKY* gene expression is influenced by a broad range of abiotic and biotic stress resistances and also responds to hormonal signals, indicating that they play a key role in signal transduction in plant resistance regulation. The role of *WRKY* genes on abiotic and biotic stress regulatory networks is systematic and complex, both between two WRKY proteins (Xu *et al.*, 2006) and their downstream or upstream targets (Jiang and Deyholos, 2009).

## Supplementary material

Supplementary data are available at *JXB* online.

Supplementary Table S1. Primers of 59 *VvWRKY* genes used for semiquantitative RT-PCR and real-time quantitative PCR.

Supplementary Table S2. WRKY domains and the characteristics of the zinc-finger motif of 59 *VvWRKY* genes.

Supplementary Table S3. The synteny regions between grape *WRKY* genes.

Supplementary Table S4. The synteny regions between grape and *Arabidopsis WRKY* genes.

Supplementary Fig. S1. Expression profiles of 59 *VvWRKY* genes under salinity stress treatment analysed using semiquantitative RT-PCR.

Supplementary Fig. S2. Expression profiles of 59 *VvWRKY* genes under drought stress treatment analysed using semiquantitative RT-PCR.

Supplementary Fig. S3. Expression profiles of 59 *VvWRKY* genes under powdery mildew (*Erysiphe necator*) inoculation analysed using semiquantitative RT-PCR.

Supplementary Fig. S4. Expression profiles of 59 *VvWRKY* genes under ABA treatment analysed using semiquantitative RT-PCR.

Supplementary Fig. S5. Expression profiles of 59 *VvWRKY* genes under SA treatment analysed using semiquantitative RT-PCR.

Supplementary Fig. S6. Expression profiles of 59 *VvWRKY* genes under MeJA treatment analysed using semiquantitative RT-PCR.

Supplementary Fig. S7. Expression profiles of 59 *VvWRKY* genes under Eth treatment analysed using semiquantitative RT-PCR.

Supplementary Data
