# Supplementary material for: Evolution and expression analysis of the grape (Vitis vinifera L.) WRKY gene family
Source: J Exp Bot. 2014 Feb 7;65(6):1513–28. doi: 10.1093/jxb/eru007 (PMC3967086; doi:10.1093/jxb/eru007)
Supplement: Supplementary Data [file supp_65_6_1513__index.html]

Evolution and expression analysis of the grape (Vitis vinifera L.) WRKY gene family — Evolution and expression analysis of the grape (Vitis vinifera L.) WRKY gene family — Supplementary Data 

# Evolution and expression analysis of the grape (*Vitis vinifera* L.) *WRKY* gene family

## Supplementary Data

Data files

**Files in this Data Supplement:**

- Supplementary Data - Supplementary Data
