# Supplementary material for: Evolution and expression analysis of the grape (Vitis vinifera L.) WRKY gene family
Source: J Exp Bot. 2014 Feb 7;65(6):1513–28. doi: 10.1093/jxb/eru007 (PMC3967086; doi:10.1093/jxb/eru007)
Supplement: Supplementary Data [file supp_eru007_jexbot110700_file001.pdf]

Evolution and Expression Analysis of the Grape (*Vitis vinifera* L.) WRKY Gene Family

Chunlei Guo [1, 2†], Rongrong Guo[1, 2†], Xiaozhao Xu[1, 2], Min Gao[1, 2], Xiaoqin Li[1, 2], Junyang Song[1, 2], Yi Zheng[3], Xiping Wang [1, 2*]

Supplementary Fig. S1. Expression profiles of 59 *VvWRKY* genes under salinity stress treatment analyzed using semi-quantitative RT-PCR. *Actin1* (GenBank Accession number AY680701) was used as an internal control. The upper and lower bands represent treatment and control, respectively

Supplementary Fig. S2. Expression profiles of 59 *VvWRKY* genes under drought stress treatment analyzed using semi-quantitative RT-PCR. *Actin1* (GenBank Accession number AY680701) was used as an internal control. The upper and lower bands represent treatment and control, respectively

Supplementary Fig. S3. Expression profiles of 59 *VvWRKY* genes under powdery mildew (*Erysiphe necator*) inoculation analyzed using semi-quantitative RT-PCR. *Actin1* (GenBank Accession number AY680701) was used as an internal control. The upper and lower bands represent treatment and control, respectively.

Supplementary Fig. S4. Expression profiles of 59 *VvWRKY* genes under ABA treatment analyzed using semi-quantitative RT-PCR. *Actin1* (GenBank Accession number AY680701) was used as an internal control. The upper and lower bands represent treatment and control, respectively.

Supplementary Fig. S5. Expression profiles of 59 *VvWRKY* genes under SA treatment analyzed using semi-quantitative RT-PCR. *Actin1* (GenBank Accession number AY680701) was used as an internal control. The upper and lower bands represent treatment and control, respectively.

Supplementary Fig. S6. Expression profiles of 59 *VvWRKY* genes under MeJA

treatment analyzed using semi-quantitative RT-PCR. *Actin1* (GenBank Accession number AY680701) was used as an internal control. The upper and lower bands represent treatment and control, respectively.

Supplementary Fig. S7. Expression profiles of 59 *VvWRKY* genes under Eth treatment analyzed using semi-quantitative RT-PCR. *Actin1* (GenBank Accession number AY680701) was used as an internal control. The upper and lower bands represent treatment and control, respectively.

Supplementary Fig. S1.

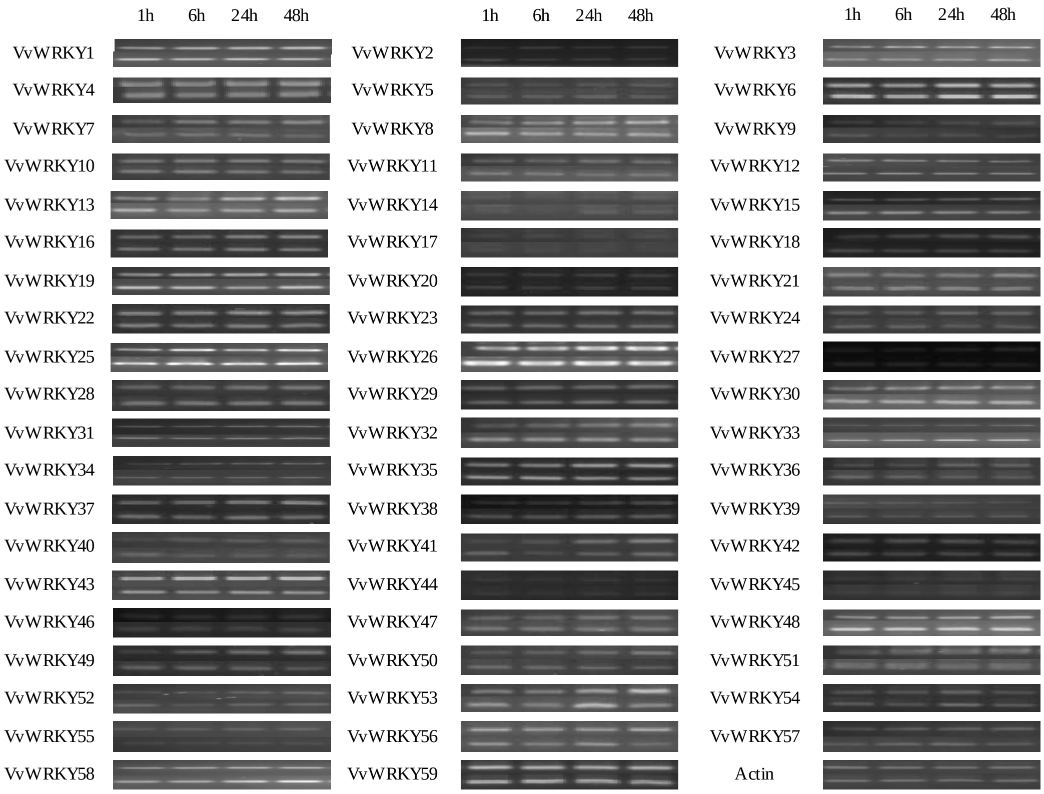

Supplementary Fig. S2.

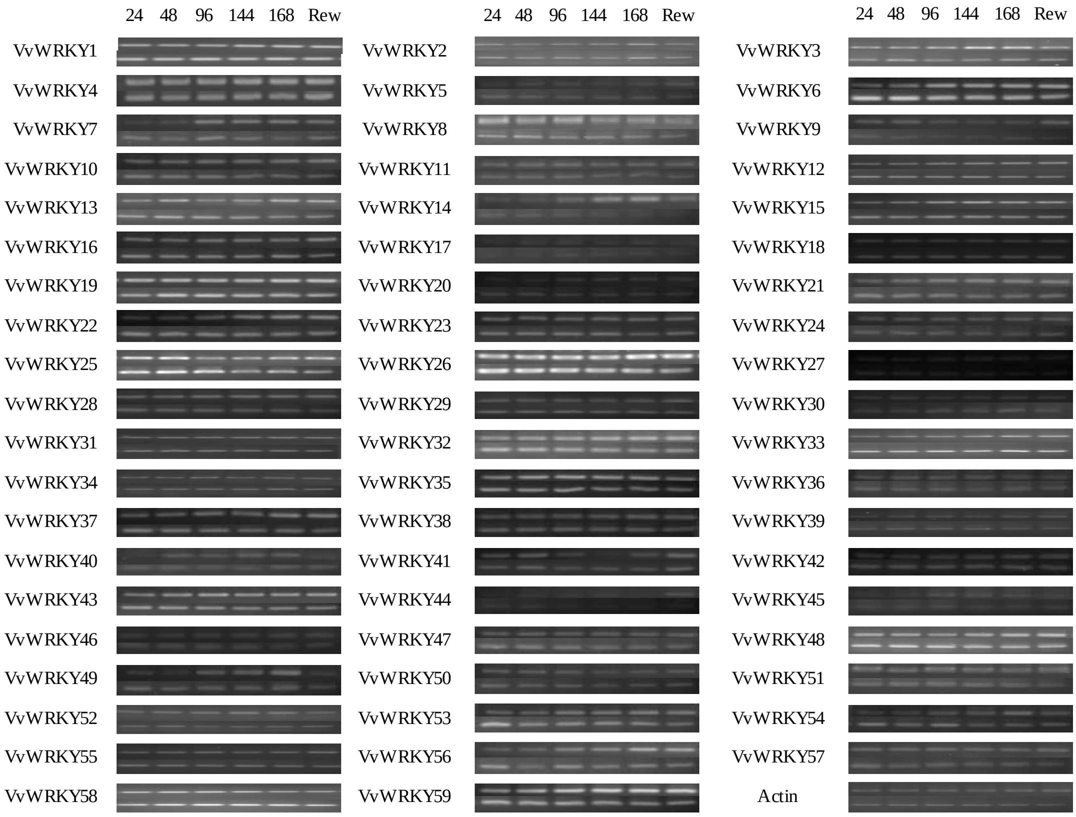

Supplementary Fig. S3.

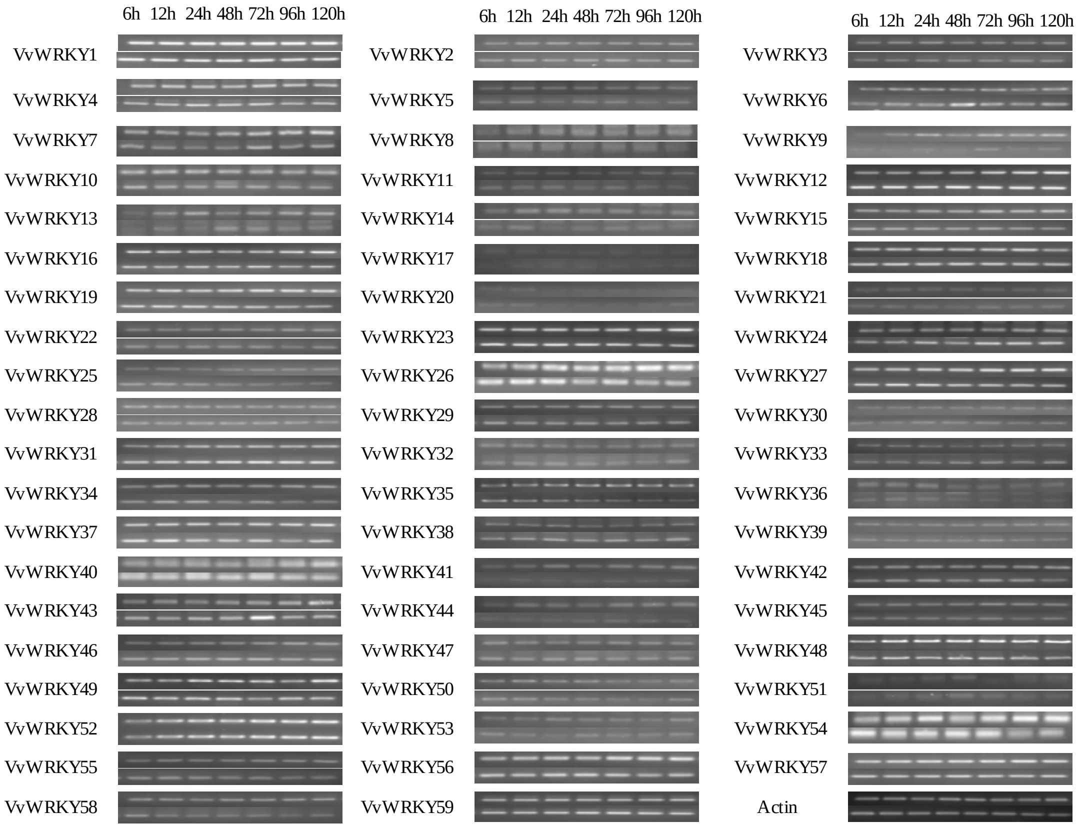

Supplementary Fig. S4.

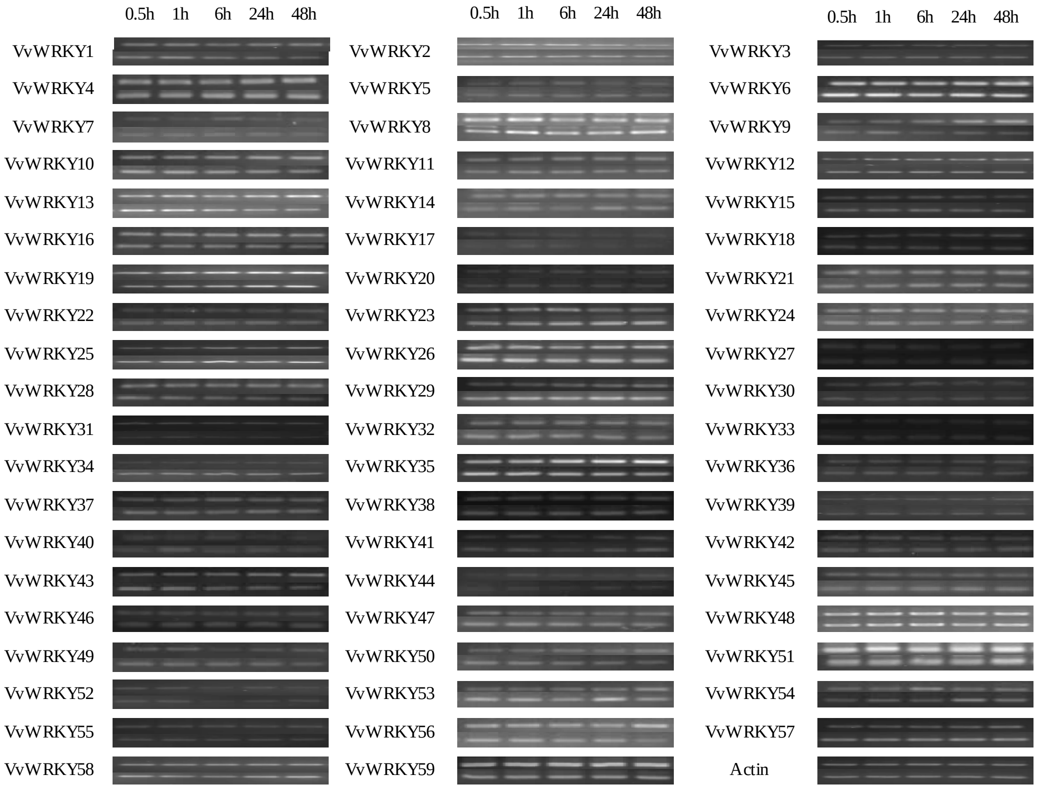

Supplementary Fig. S5.

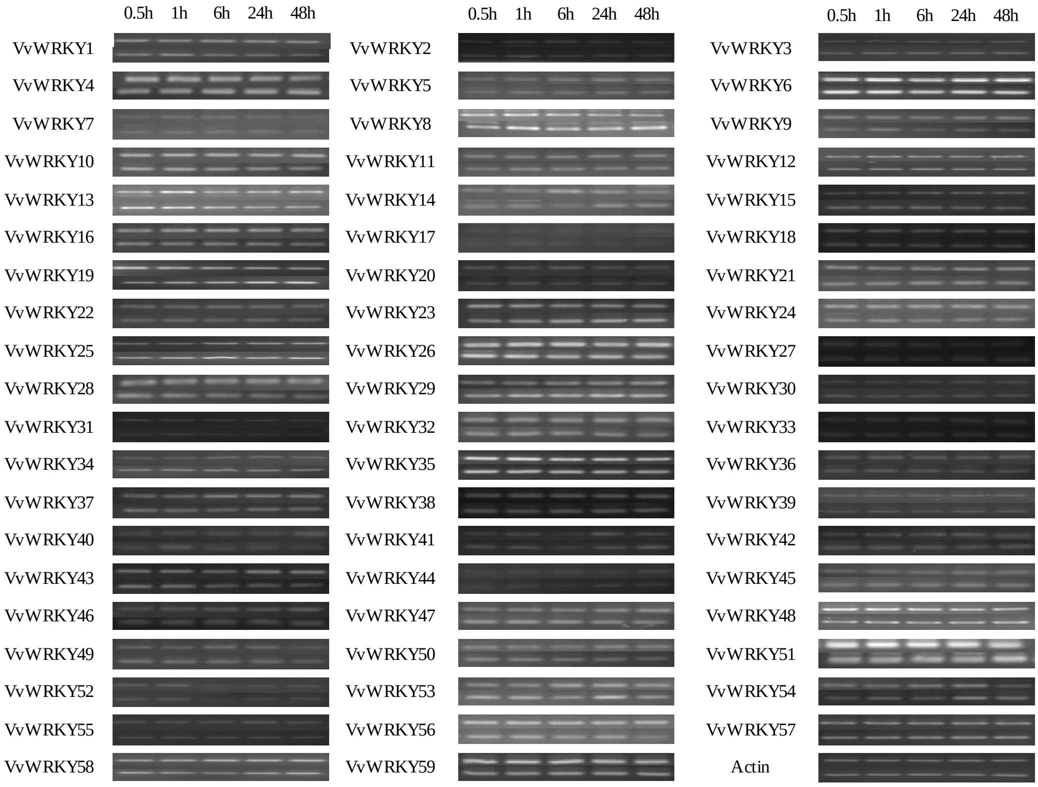

Supplementary Fig. S6.

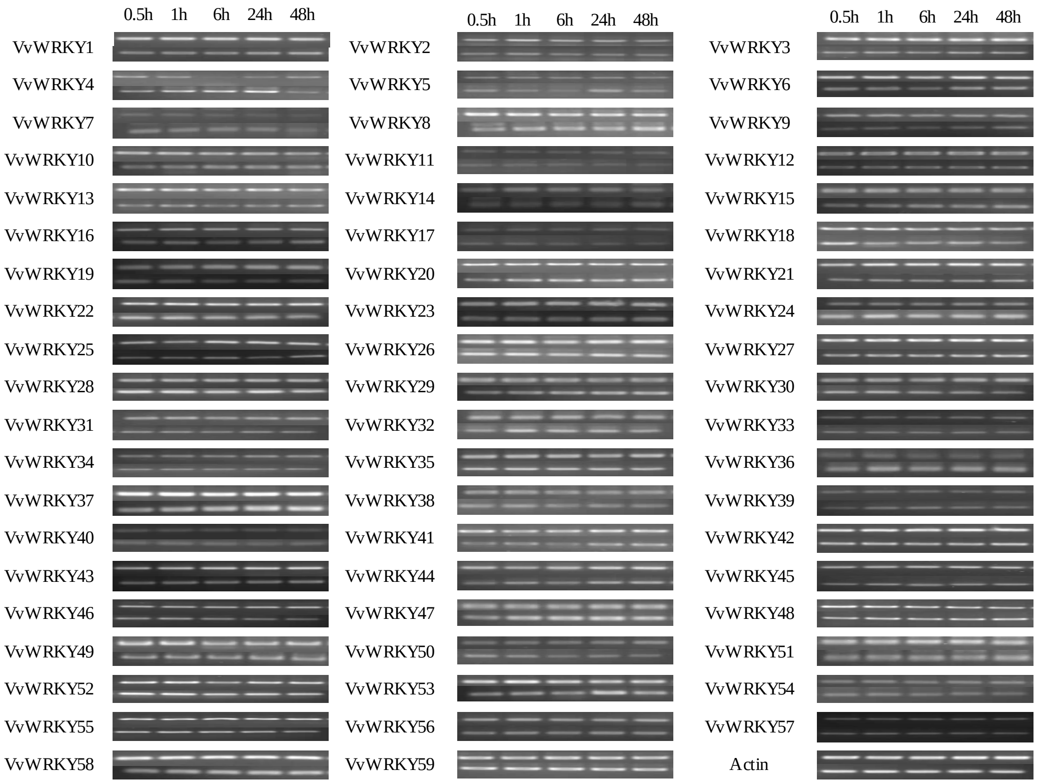

Supplementary Fig. S7.

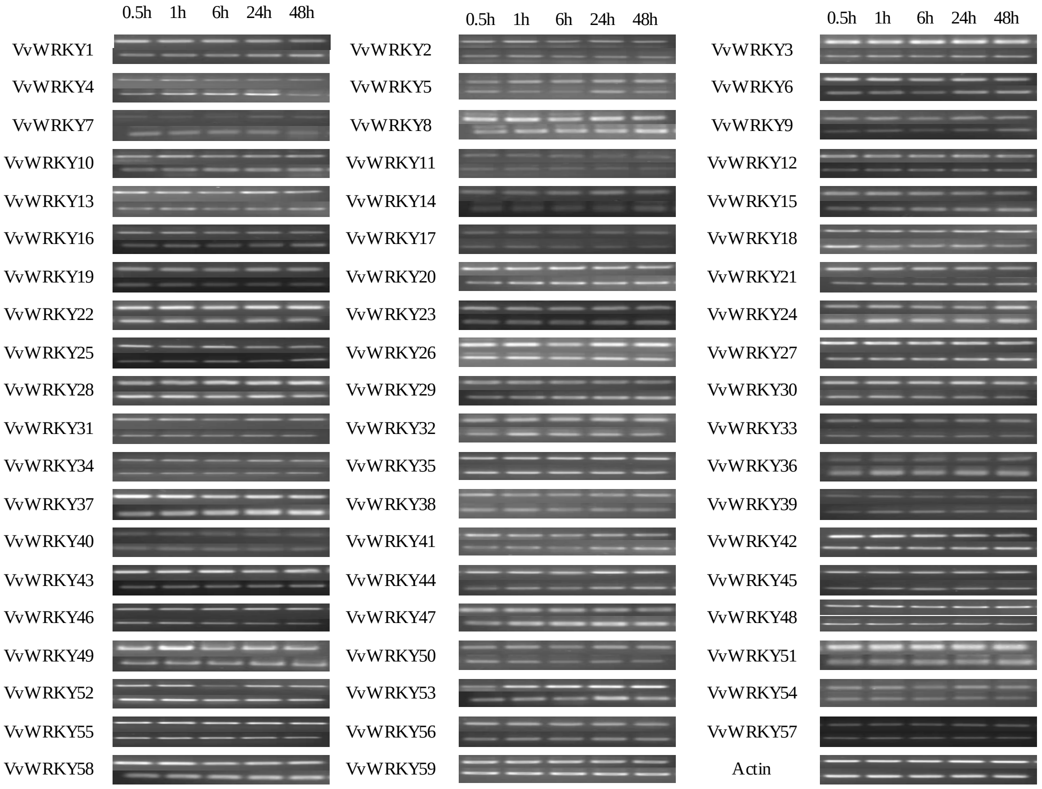

Supplementary Table S1. Primers of 59 *VvWRKY* genes

Gene ID	Gene locus ID	Primers	
		Forward primer(5’ to 3’)	Reverse primer(5’ to 3’)
VvWRKY1	GSVIVT01012196001	GACCCCTCCATTAACATCACA	CCCAAGCAGTCCTTCATCAGT
VvWRKY2	GSVIVT01020060001	TATGGGGCACTGTCGTCCT	GTTGCGGTGGCTATGGTC
VvWRKY3	GSVIVT01010525001	AGTTCCCGCCTCAGACATC	TTTTGCCCATACTTCCTCC
VvWRKY4	GSVIVT01001332001	ATCAAGCACCACTACCCA	CTGAAACCCTGACCTCTG
VvWRKY5	GSVIVT01019419001	TGTGGGCTTGGCGTAAATA	GGCGAGTTGGATGAGAATGG
VvWRKY6	GSVIVT01019511001	AAACAGAACCACCATAACGACA	GGAGAATAACAGCCCAAGAGG
VvWRKY7	GSVIVT01001286001	AGGAAATACGGGCAAAAG	GCAGAACAGAGTCCCACAAA
VvWRKY8	GSVIVT01035426001	ATTCCTGGCATCCGAGTTT	CTTGGATTTGGGCTGTTCTT
VvWRKY9	GSVIVT01035884001	CGGGACGGGTCAGGAGAAG	CGCAGCAGCCAAAGCAAG
VvWRKY10	GSVIVT01035885001	GAAAGTATGGGCAGAAGGTCAC	ACTGGTGAAGGTTGCTGGT
VvWRKY11	GSVIVT01035965001	TAAGAAGCGGCATAGTCCAC	TCTCACCTCCAAGTTCCAA
VvWRKY12	GSVIVT01033188001	GGTTTCTTCTTCTGATTCTCCCTC	AGATGACCCCTGCTTTCCGT
VvWRKY13	GSVIVT01033194001	GCCAAGTGAAGAAGAGGATAGA	AAGGAGATTGGGAGGAAGC
VvWRKY14	GSVIVT01033195001	GATGCCAAGTGAAGAAGAGG	TGTGATGTGGTTGCGAGGG

VvWRKY15	GSVIVT01019109001	TGCTGATCCCCACCAACC	CTGCTGCCCCACGAAATA
VvWRKY16	GSVIVT01034968001	TGGCTAATTCAACATTTTCCGA	TTCACAACCTCCGACGACT
VvWRKY17	GSVIVT01025491001	GACAAATCAACAACCCCACTT	CCCACCTCCCAGGAAAAC
VvWRKY18	GSVIVT01024624001	TGGCAGTTACCACGCATCA	CTCGGGTTTTCGCTTCCTT
VvWRKY19	GSVIVT01000752001	GGGGAGGCTGTGGTTAGGTT	GTTTGGCATTTGGCTTGTCT
VvWRKY20	GSVIVT01028129001	GGTCTTGGAGGAAATATGGG	TAGGCTGTTGGGAGTGGT
VvWRKY21	GSVIVT01028147001	TCGTCGGCGTCCAGTCAT	ATCAACCTCGCTCTTTGTCA
VvWRKY22	GSVIVT01028244001	CAAGCCAGGGACCAGACA	GACATCACCTCCGTAGTATTAGC
VvWRKY23	GSVIVT01022067001	CAAAGCGAGGAATCACAGGG	CAGCGACGACATAAACGAA
VvWRKY24	GSVIVT01022245001	TTTGAACCCTCCGACTACC	CTTCGCCCCATCTTTACAT
VvWRKY25	GSVIVT01022259001	GGTGGATGTTCTTGATGACGG	GTGGGAGGGCGAATGAATGTG
VvWRKY26	GSVIVT01030258001	GACCTCTTGGACTCCCCTC	AAGCCTGCTGGTGCTCGTT
VvWRKY27	GSVIVT01030174001	CTTGGATCAGAATCACCCCTAA	GCCGTGGTATGTGGTTTTGTA
VvWRKY28	GSVIVT01025562001	TTCTGGGTGATGGCTTTCG	CTGGGTCCTCTGATGCTCT
VvWRKY29	GSVIVT01034148001	AGGCTATTAGTCGGCTGGTAT	ATTGAGGTTCTGGCTTGTGA
VvWRKY30	GSVIVT01015952001	TTGGGGTTGAAGGTCGTCT	TCTCGGGGTTCTTGCTGAT
VvWRKY31	GSVIVT01012682001	CTCACCCTTGATTCTATTGCC	AAATCCACCTCCCGCCTC

VvWRKY32	GSVIVT01021252001	CTCGCCACCTTCTCCTATGT	TGGGCACTGACACCACTCT
VvWRKY33	GSVIVT01021397001	TTTGGCTTGTCACCTTCATCC	CGGTCCTTCTTACTCTTGCTT
VvWRKY34	GSVIVT01021765001	GTGCGTCTGTGAAAGAGGAG	GCGAACAATAGATTTAGAGGGT
VvWRKY35	GSVIVT01023600001	CTAAACGGAGATCGAAGAAAAG	TACGAACAGGACATCCAGCA
VvWRKY36	GSVIVT01029265001	CCTCTTTCGTCGTCTCAAC	GCTTATCGCAGGAACTCT
VvWRKY37	GSVIVT01020864001	CAGTCAAGAATAGCCCTTACCC	CAGCATTGCCTCTTATCGTAG
VvWRKY38	GSVIVT01030453001	AGGTTCCCAGGTTGAATCCC	CTTTGCCATCTTCTGCCCAT
VvWRKY39	GSVIVT01030046001	GCCACTCCGACCGATAAGC	CCTTGGATTTGTATTGCCTTT
VvWRKY40	GSVIVT01029688001	ACCAAATCCTTCCATCATCAC	TGCAGCCAAGTCAGTCCTAG
VvWRKY41	GSVIVT01032662001	GAACCTGTGACCTTGTCGG	CCTTTCTTCTTCGTAATCTTTGC
VvWRKY42	GSVIVT01032661001	GAGGAAGCATGAGCAAAGC	GAATCATAGCCAGGATAAGAGC
VvWRKY43	GSVIVT01036223001	AAAGCCAAAATCAAGCCTCC	GTCCAAGTTAGCAACACTACCATC
VvWRKY44	GSVIVT01033063001	TTCCAATGTTTTCGGTAATGT	CAAAGCCTCCACTGCTCCTA
VvWRKY45	GSVIVT01011356001	GCCTCCCAGTAAAGTTTTG	ATCATTCATCGTTAGCGTGTC
VvWRKY46	GSVIVT01011472001	TACCATCAGACCCTAAGAGTTCC	TGTCCATATTTCCGCCAGTT
VvWRKY47	GSVIVT01018300001	GGAATAATGAGCAGGTGGGG	ACTTTGGTCTTTTCTGAGGATG
VvWRKY48	GSVIVT01027069001	CAAGATTTCAAGGACCAAGCAG	AGTATGCCTTCCTCGGTATGT

VvWRKY49	GSVIVT01026969001	CCCACATCTTCAACTCCCACC	TGATGCTGCCCTTTTCTTCC
VvWRKY50	GSVIVT01026965001	CTCTGTGCCCTCCACTACG	AACACCCCTTTGAACTGCTAC
VvWRKY51	GSVIVT01028823001	AGAATGAAAACGAAAACAGTGAG	GCAATGGTCGGTTGTGAAT
VvWRKY52	GSVIVT01028718001	CCTCTTGATGATGGGTTTAGTT	GTCTTCCACGGTAGGTGATTT
VvWRKY53	GSVIVT01008553001	CGATGGAGGAAATATGGGC	TGTTCAAAGTTCTCGGTGGG
VvWRKY54	GSVIVT01008046001	AACAAAGGCAATCGCATCA	ATAGGTAGAAACAGCAGGAAGG
VvWRKY55	GSVIVT01009441001	GGAGACACCGAAAGCGT	AATGACACCGCCCAAAAG
VvWRKY56	GSVIVT01037686001	ATGCTACTTTCGGGATCAATG	TCTGGTGCTGTAAGAGGTTCG
VvWRKY57	GSVIVT01037775001	GCAGGACAAGTAGCTCTGAA	TAGTGCGGGTTTGGGTTAG
VvWRKY58	GSVIVT01014854001	GAGGTCTTGTGGAAAGGATAGC	CCAGGAGGGATTGTCAGGTA
VvWRKY59	GSVIVT01007006001	TGTTACTGCTCCCAATCCAC	GCCCATACTTCCGCCAAT

Supplementary Table S2. WRKY domains and the characteristic of zinc-finger motif of 59 *VvWRKY* genes

Gene ID	Gene WRKY domain		
	Conserved heptapeptide	Domain number	Zinc-finger motif
VvWRKY1	WRKYGQK	1	C2H2/ C2H2
VvWRKY2	WRKYGQK	1	C2H2
VvWRKY3	WRKYGQK	1	C2H2
VvWRKY4	WRKYGQK/ WRKYGQK	2	C2H2/ C2H2
VvWRKY5	WRKYGQK	1	C2H2
VvWRKY6	WRKYGQK	1	C2HC
VvWRKY7	WRKYGQK	1	-C--
VvWRKY8	WRKYGKK	1	C2H2
VvWRKY9	WRKYGQK	1	C2H2
VvWRKY10	WRKYGQK	1	C2H2
VvWRKY11	WRKYGQK/ WRKYGQK	2	C2H2/ C2H2
VvWRKY12	WRKYGQK	1	C2H2
VvWRKY13	WRKYGKK	1	C2H2
VvWRKY14	WRKYGKK	1	C2H2
VvWRKY15	WRKYGQK/ WRKYGQK	2	C2H2/ C2H2
VvWRKY16	WRKYGQK	1	C2H2
VvWRKY17	WKKYGQK	1	--
VvWRKY18	WRKYGQK/ WRKYGQK	2	C2H2/ C2H2
VvWRKY19	WRKYGQK	1	C2H2
VvWRKY20	WRKYGQK	1	C2H2
VvWRKY21	WRKYGQK	1	C2H2
VvWRKY22	WRKYGQK	1	C2H2
VvWRKY23	WRKYGQK	1	C2H2
VvWRKY24	WRKYGKK	1	C2H2
VvWRKY25	WRKYGQK	1	C2H2

VvWRKY26	WRKYGQK/ WRKYGQK	2	C2H2/ C2H2
VvWRKY27	WRKYGQK	1	C2HC
VvWRKY28	WRKYGQK/ WRKYGQK	2	C2H2/ C2H2
VvWRKY29	WRKYGQK	1	C2H2
VvWRKY30	WRKYGQK	1	C2H2
VvWRKY31	WRKYGQK	1	C2H2
VvWRKY32	WRKYGQK	1	C2H2
VvWRKY33	WRKYGQK	1	C2H2
VvWRKY34	WRKYGQK	1	C2H2
VvWRKY35	WRKYGQK/ WRKYGQK	2	C2H2/ C2H2
VvWRKY36	WRKYGQK	1	C2H2
VvWRKY37	WRKYGQK	1	C2H2
VvWRKY38	WRKYGQK	1	C2H2
VvWRKY39	WRKYGQK/ WRKYGQK	2	C2H2/ C2H2
VvWRKY40	WRKYGQK	1	C2H2
VvWRKY41	WRKYGQK	1	C2HC
VvWRKY42	WRKYGQK	1	C2HC
VvWRKY43	WRKYGQK	1	C2H2
VvWRKY44	WRKYGQK	1	C2H2
VvWRKY45	WRKYGQK	1	C2H2
VvWRKY46	WRKYGQK/ WRKYGQK	2	--/C2H2
VvWRKY47	WRKYGQK	1	C2H2
VvWRKY48	WRKYGQK	1	C2HC
VvWRKY49	WRKYGQK	1	C2H2
VvWRKY50	WRKYGQK	1	C2H2
VvWRKY51	WRKYGQK	1	C2H2
VvWRKY52	WRKYGQK	1	C2HC
VvWRKY53	WRKYGQK	1	C2H2
VvWRKY54	WRKYGQK	1	C2H2

VvWRKY55	WRKYGQK	1	C2H2
VvWRKY56	WRKYGQK	1	C2H2
VvWRKY57	WRKYGQK/ WRKYGQK	2	C2H2/ C2H2
VvWRKY58	WRKYGQK/ WRKYGQK	2	C2H2/ C2H2
VvWRKY59	WRKYGQK/ WRKYGQK	2	C2H2/ C2H2

Colored conserved heptapeptides were those *VvWRKY* genes different compared with other genes.

Supplementary Table S3. The synteny regions between grape WRKY genes

ID	region 1 (Grape gene 1)			region2 (Grape gene 2)			gene in the synteny region		gene 1_name	gene 2_name
	Chr	Start	Stop	Chr	Start	Stop	gene 1	gene 2		
1	chr17	5814755	7662326	chr14	28457051	30137019	GSVIVT01008046001	GSVIVT01011356001	*VvWRKY54*	*VvWRKY45*
2	chr17	5814755	7784744	chr1	10067645	15346615	GSVIVT01008046001	GSVIVT01020060001	*VvWRKY54*	*VvWRKY2*
3	chr18	8368864	9355161	chr7	16300878	17030189	GSVIVT01009441001	GSVIVT01022067001	*VvWRKY55*	*VvWRKY23*
4	chr1	21240516	22853689	chr14	25536232	24482249	GSVIVT01010525001	GSVIVT01033063001	*VvWRKY3*	*VvWRKY44*
5	chr14	28844329	29813645	chr1	10815152	13910631	GSVIVT01011356001	GSVIVT01020060001	*VvWRKY45*	*VvWRKY2*
6	chr10	414663	971082	chr12	6054656	5480518	GSVIVT01012682001	GSVIVT01030453001	*VvWRKY31*	*VvWRKY38*
7	chr10	503092	1108943	chr19	6964793	6519950	GSVIVT010126	GSVIVT010376	*VvWRKY31*	*VvWRKY56*

							82001	86001		
8	chr2	236398	2725052	chr15	19083025	16064252	GSVIVT01019419001	GSVIVT01026965001	*VvWRKY5*	*VvWRKY50*
9	chr2	308882	1535757	chr16	17905499	20888112	GSVIVT01019419001	GSVIVT01028823001	*VvWRKY5*	*VvWRKY51*
10	chr6	7674586	8985737	chr8	8767790	10972180	GSVIVT01024624001	GSVIVT01030258001	*VvWRKY18*	*VvWRKY26*
11	chr15	17436787	19057628	chr16	20816151	18064705	GSVIVT01026965001	GSVIVT01028823001	*VvWRKY50*	*VvWRKY51*
12	chr7	3200984	4418032	chr5	1809826	249313	GSVIVT01028147001	GSVIVT01034968001	*VvWRKY21*	*VvWRKY16*
13	chr12	9017193	9747379	chr19	7643696	7903218	GSVIVT01030046001	GSVIVT01037775001	*VvWRKY39*	*VvWRKY57*
14	chr8	7406578	10869614	chr13	501561	1751444	GSVIVT01030174001	GSVIVT01032661001	*VvWRKY27*	*VvWRKY42*
15	chr12	5480518	6585356	chr19	6671393	7347760	GSVIVT01030453001	GSVIVT01037686001	*VvWRKY38*	*VvWRKY56*

Supplementary Table S4. The synteny regions between grape and *Arobidopsis* WRKY genes

ID	region 1 (Arobidopsis gene 1)			region2 (Grape gene 2)			gene in the synteny region		grape gene_name
	Chr	Start	Stop	Chr	Start	Stop	gene 1	gene 2	
1	Chr1	26274230	26335558	chr1	10987218	10323462	AT1G69810	GSVIVT01020060001	*VvWRKY2*
2	Chr1	10323655	10920677	chr10	4613342	10010600	AT1G29860	GSVIVT01021397001	*VvWRKY33*
3	Chr1	9911898	10266818	chr10	1335630	3199699	AT1G29280	GSVIVT01021252001	*VvWRKY32*
4	Chr1	22882215	23044855	chr10	1118628	433319	AT1G62300	GSVIVT01012682001	*VvWRKY31*
5	Chr1	22932901	23104647	chr12	5491822	6546456	AT1G62300	GSVIVT01030453001	*VvWRKY38*
6	Chr1	26063816	26626503	chr14	30011357	27051487	AT1G69810	GSVIVT01011356001	*VvWRKY45*
7	Chr1	23515874	23866045	chr15	19693047	18339430	AT1G64000	GSVIVT01026969001	*VvWRKY49*
8	Chr1	6426898	6560489	chr17	7218050	5809177	AT1G18860	GSVIVT01008046001	*VvWRKY54*
9	Chr1	26050197	26527345	chr17	8115895	5482657	AT1G69810	GSVIVT01008046001	*VvWRKY54*
10	Chr1	22824440	23109476	chr19	6153028	7268617	AT1G62300	GSVIVT01037686001	*VvWRKY56*
11	Chr1	30311666	30385499	chr4	5744083	5247592	AT1G80840	GSVIVT01035884001	*VvWRKY9*
12	Chr1	30246745	30404690	chr9	16000783	16883252	AT1G80840	GSVIVT01015952001	*VvWRKY30*
13	Chr2	1698466	1934212	chr12	8990937	9698217	AT2G04880	GSVIVT01030046001	*VvWRKY39*

14	Chr2	16700510	17021315	chr13	185617	1775361	AT2G40740	GSVIVT01032662001	*VvWRKY41*
15	Chr2	18379564	18853709	chr15	9935686	16207483	AT2G44745	GSVIVT01018300001	*VvWRKY47*
16	Chr2	18855775	19277773	chr15	20079735	16362838	AT2G46130	GSVIVT01026969001	*VvWRKY49*
17	Chr2	9513219	9946269	chr18	5092140	8531571	AT2G23320	GSVIVT01009441001	*VvWRKY55*
18	Chr2	18991171	19085704	chr2	838383	1382859	AT2G46400	GSVIVT01019511001	*VvWRKY6*
19	Chr2	10629737	10745594	chr4	5247592	5744083	AT2G25000	GSVIVT01035884001	*VvWRKY9*
20	Chr2	10629737	10972729	chr4	5248886	3888015	AT2G25000	GSVIVT01035884001	*VvWRKY9*
21	Chr2	11921433	13011063	chr6	3098092	9133237	AT2G30250	GSVIVT01024624001	*VvWRKY18*
22	Chr2	9504659	9975192	chr7	14791101	16682195	AT2G23320	GSVIVT01022067001	*VvWRKY23*
23	Chr2	13019028	13078595	chr7	323141	508699	AT2G30590	GSVIVT01000752001	*VvWRKY19*
24	Chr2	19277903	19696821	chr7	4750345	1588071	AT2G47260	GSVIVT01028147001	*VvWRKY21*
25	Chr2	9281986	9460919	chr7	18225477	17139552	AT2G21900	GSVIVT01022245001	*VvWRKY24*
26	Chr2	12781591	12985043	chr8	7693491	10767781	AT2G30250	GSVIVT01030258001	*VvWRKY26*
27	Chr2	16886824	17142457	chr8	10305172	11100975	AT2G40750	GSVIVT01030174001	*VvWRKY27*
28	Chr2	15576024	15705346	chr8	14204857	13626007	AT2G37260	GSVIVT01025562001	*VvWRKY28*
29	Chr2	16072070	16111797	chr8	10373424	9793737	AT2G38470	GSVIVT01030258001	*VvWRKY26*
30	Chr3	20714946	21034455	chr13	156285	2110460	AT3G56400	GSVIVT01032662001	*VvWRKY41*

31	Chr3	9793220	11269229	chr14	24473557	30137019	AT3G29185	GSVIVT01011472001	*VvWRKY46*
32	Chr3	1146800	1277824	chr14	7336891	8775630	AT3G04670	GSVIVT01036223001	*VvWRKY43*
33	Chr3	1653	350010	chr14	30170199	25221541	AT3G01970	GSVIVT01033063001	*VvWRKY44*
34	Chr3	23023437	23186701	chr7	4674928	3351619	AT3G62340	GSVIVT01028147001	*VvWRKY21*
35	Chr3	20780315	21041760	chr8	9483459	11436191	AT3G56400	GSVIVT01030174001	*VvWRKY27*
36	Chr4	10016567	10290850	chr10	4024027	6838091	AT4G18170	GSVIVT01021397001	*VvWRKY33*
37	Chr4	1870422	2429850	chr10	256137	1176476	AT4G04450	GSVIVT01012682001	*VvWRKY31*
38	Chr4	11532971	11719828	chr10	1298924	433319	AT4G22070	GSVIVT01012682001	*VvWRKY31*
39	Chr4	14847740	15110289	chr11	13795524	7317082	AT4G30935	GSVIVT01023600001	*VvWRKY35*
40	Chr4	11655589	11792876	chr12	5491822	6531111	AT4G22070	GSVIVT01030453001	*VvWRKY38*
41	Chr4	1887528	2330200	chr12	6327193	5491822	AT4G04450	GSVIVT01030453001	*VvWRKY38*
42	Chr4	431963	543869	chr15	17990229	19057628	AT4G01250	GSVIVT01026965001	*VvWRKY50*
43	Chr4	11963628	12393982	chr15	19643685	18191021	AT4G23550	GSVIVT01026965001	*VvWRKY50*
44	Chr4	11613314	11810117	chr19	6453337	7517479	AT4G22070	GSVIVT01037686001	*VvWRKY56*
45	Chr4	13437071	13761559	chr19	7767468	2128359	AT4G26640	GSVIVT01037775001	*VvWRKY57*
46	Chr4	12387840	12539387	chr2	1164898	2335451	AT4G23810	GSVIVT01019511001	*VvWRKY6*
47	Chr4	12240803	12302719	chr2	209926	544068	AT4G23550	GSVIVT01019419001	*VvWRKY5*

48	Chr4	6696712	6777225	chr2	1627954	1066670	AT4G11070	GSVIVT01019511001	*VvWRKY6*
49	Chr4	15383201	15500847	chr4	5247592	5930252	AT4G31800	GSVIVT01035884001	*VvWRKY9*
50	Chr4	700653	795139	chr7	4682950	5000938	AT4G01720	GSVIVT01028244001	*VvWRKY22*
51	Chr4	18293129	18471967	chr7	18225477	17118430	AT4G39410	GSVIVT01022259001	*VvWRKY25*
52	Chr5	18707646	18886939	chr10	6748051	4018498	AT5G46350	GSVIVT01021397001	*VvWRKY33*
53	Chr5	4477354	5057411	chr14	24525304	30253071	AT5G15130	GSVIVT01011356001	*VvWRKY45*
54	Chr5	4149740	4307513	chr14	25481683	24792503	AT5G13080	GSVIVT01033063001	*VvWRKY44*
55	Chr5	16503774	16709909	chr15	19612387	18344707	AT5G41570	GSVIVT01026969001	*VvWRKY49*
56	Chr5	20797017	21643562	chr16	8000259	19401729	AT5G52830	GSVIVT01028823001	*VvWRKY51*
57	Chr5	8005234	8369866	chr16	16867341	21295918	AT5G24110	GSVIVT01028718001	*VvWRKY52*
58	Chr5	4889295	4976227	chr17	6167656	6995184	AT5G15130	GSVIVT01008046001	*VvWRKY54*
59	Chr5	4149740	4274660	chr17	925171	108833	AT5G13080	GSVIVT01008553001	*VvWRKY53*
60	Chr5	22121404	22792161	chr19	4954907	10830108	AT5G56270	GSVIVT01014854001	*VvWRKY58*
61	Chr5	9137461	9160383	chr4	1115013	1296907	AT5G26170	GSVIVT01035426001	*VvWRKY8*
62	Chr5	20080614	20312808	chr5	542600	262046	AT5G49520	GSVIVT01034968001	*VvWRKY16*
63	Chr5	2200332	2266967	chr6	8442467	7674586	AT5G07100	GSVIVT01024624001	*VvWRKY18*
64	Chr5	19953487	20139384	chr7	3495915	4392246	AT5G49520	GSVIVT01028147001	*VvWRKY21*

65	Chr5	25872346	26048031	chr7	18225477	17324619	AT5G64810	GSVIVT01022245001	*VvWRKY24*
66	Chr5	129302	362901	chr8	8219718	10911693	AT5G01900	GSVIVT01030174001	*VvWRKY27*
